# Time wears on: Assessing how bone wears using 3D surface texture analysis

**DOI:** 10.1371/journal.pone.0206078

**Published:** 2018-11-07

**Authors:** Naomi L. Martisius, Isabelle Sidéra, Mark N. Grote, Teresa E. Steele, Shannon P. McPherron, Ellen Schulz-Kornas

**Affiliations:** 1 Department of Anthropology, University of California Davis, Davis, CA, United States of America; 2 Maison Archéologie et Ethnologie René-Ginouvès, Centre National de la Recherche Scientifique, Université Paris Ouest Nanterre, Nanterre, France; 3 Department of Human Evolution, Max Planck Institute for Evolutionary Anthropology, Leipzig, Germany; 4 Max Planck Weizmann Center for Integrative Archaeology and Anthropology, Max Planck Institute for Evolutionary Anthropology, Leipzig, Germany; Monash University, AUSTRALIA

## Abstract

Use-wear analysis provides a means of studying traces produced on animal bone during manufacture and use in an effort to reconstruct these processes. Often, these analyses are qualitative and based on experience and expertise. Previous studies have focused on interpreting final traces, but little is known about how these traces develop and change over time. We propose the use of an innovative quantitative method for studying bone surface traces that aims to reduce any unreliable or non-replicable results that can confound more traditional qualitative analyses. We seek to understand the basics of use-wear formation over *Time* by taking incremental molds of bone specimens subjected to a controlled, mechanical experiment. This study assesses how bone wears during extended use on three *Material types* (fresh skin, processed leather, or dry bark), from three initial *Manufacturing states* (unworked, ground with sandstone, or scraped with flint). With data obtained from a confocal disc-scanning microscope, we then apply 3D surface texture analysis using ISO 25178 parameters: surface roughness [*Sa*], autocorrelation length [*Sal*], peak curvature [*Spc*], and upper material ratio [*Smr1*]. We employ a multilevel multivariate Bayesian model to explain parameter variation under experimental conditions. Our findings show how duration of use strongly affects the transformation of the bone’s surface. Unworked bone is completely distinguishable from bone used for long time intervals and those modified by scraping. Interestingly, material wear does not often produce type-specific traces, but does affect the rate of bone alteration and how it is transformed. Specifically, fresh skin transforms bone at a faster rate than other materials. This novel quantitative and experimental approach enhances our understanding of the use of bone as a raw material for making and using tools and provides a foundation for future exploration of archaeological materials and questions.

## Introduction

Bone tool use has been documented as early as 2 million years ago (mya) in the hominin record [1, 2]. These cases represent expedient tools and are recognizable by the wear traces produced during use. As early as 1.4 mya, hominins began to modify bones in a manner similar to that in which they knapped stone [3]. By approximately 106 thousand years ago, hominins in North Africa were producing formal tools that used manufacturing techniques specific to bone such as scraping and grinding [4–7], and relatively soon after, various types of formally manufactured bone tools became more frequently used in other parts of the African continent [8–10]. The techniques used for shaping bone into a variety of objects became widespread throughout various geographic locations and time periods thereafter (see *e*.*g*., [11–18]).

Wear traces on bone produced by shaping during manufacture and by use have been studied within in the field of use-wear analysis with theoretical underpinnings derived from tribology, the study of how two surfaces in relative motion interact with each other. This approach, most often used for industrial purposes, focuses on the study of lubrication, friction, and wear [19, 20]. Archaeologists have adapted tribology to understand the patterns of wear found on artifacts and many have carried out research devoted specifically to developing the field of use-wear analysis on bone tools [19, 21–27]. Traditionally, use-wear analyses on bone have been qualitative in nature using light microscopy or scanning electron microscopy [19, 24, 26–32]. Interpretations of the use-wear traces are typically based on experience and expertise and less frequently on explicit protocols. This can produce inconsistencies and unreplicable results [27].

Most use-wear studies have focused on interpreting the final traces on a tool, but little is known about the development of these traces and how they change over time. Traces on a given material may be completely different depending on the duration or action of use [29]. Similarly, objects minimally used on different materials may exhibit similar traces. Different states of a particular material (*e*.*g*., fresh versus raw versus softened animal hide) may also confound results and be difficult to distinguish. Use-wear traces may take on different forms based on characteristics of the raw material, such as the natural microtopography of the bone surface itself [23, 30]. For these reasons, the development of a quantitative methodology for studying bone tools that produces replicable results is essential.

Research on bone surface modifications has been enhanced by the use of quantitative 3D methods [33–38]. These approaches and others that utilize confocal microscopy and 3D surface texture analysis [39] have appreciably reduced intra-observer error. 3D surface texture analysis was originally developed in the field of surface metrology [40], but it has been adapted for anthropological, biological, and archaeological applications in wear studies on teeth [41–46] and lithics [47–55], and less frequently on ochre [56] and bone tools [57–59].

3D quantitative methods to understand archaeological materials is largely experimental in nature. Experimental investigation can help us understand past processes observed in archaeological contexts because it creates empirical observations of known conditions that can then be applied using analogy to questions derived from the archaeological record [60–63]. In the 1960s and 70s, within the “middle-range” framework, archaeologists focused on linking the archaeological record with the dynamic processes responsible for creating it [64–67]. In recent years, archaeologists have focused on question-based experimentation in order to test hypotheses and generate predictions related to replicative experiments [68], and they are conducting controlled lab-based experiments designed to accurately understand the variation in the dependent variables established at the start of the experiment [69–71].

Our study represents an effort to integrate both prediction-based and controlled, replicable experiments to understand the basics of use-wear formation over time and to develop a quantitative methodology for measuring the microtopography of bone surfaces. The experiment was not designed to assess the range of all possibilities for understanding bone use and manufacture. Here, we chose to increase external and internal validity and reduce ecological validity in order to focus on a select number of independent variables [72]. The external validity of this experiment derives from its broad focus on the development of use-wear on bone. The experiment was not designed to understand wear formation on a specific technological type coming from a specific location at a specific time period, but to understand how bone wears through time in general. The results of this type of experiment are then applicable to a wide variety of other bone use-wear studies. The internal validity of the experiment comes from the mechanical setup, which allows for control of a number of variables (*e*.*g*., time, motion, and strokes per minute) on the machine itself, while our sampling procedure could be standardized and replicated. By controlling these variables in laboratory conditions, we were able to focus on the effects of variation in other selected independent variables (*Time*, *Manufacturing state*, *Material type*) to address questions of tool manufacture, use, and wear patterns. The experimental setup will allow for the development of our quantitative approach and on eventual translation to the archaeological record.

## Materials and methods

**Fig 1:**
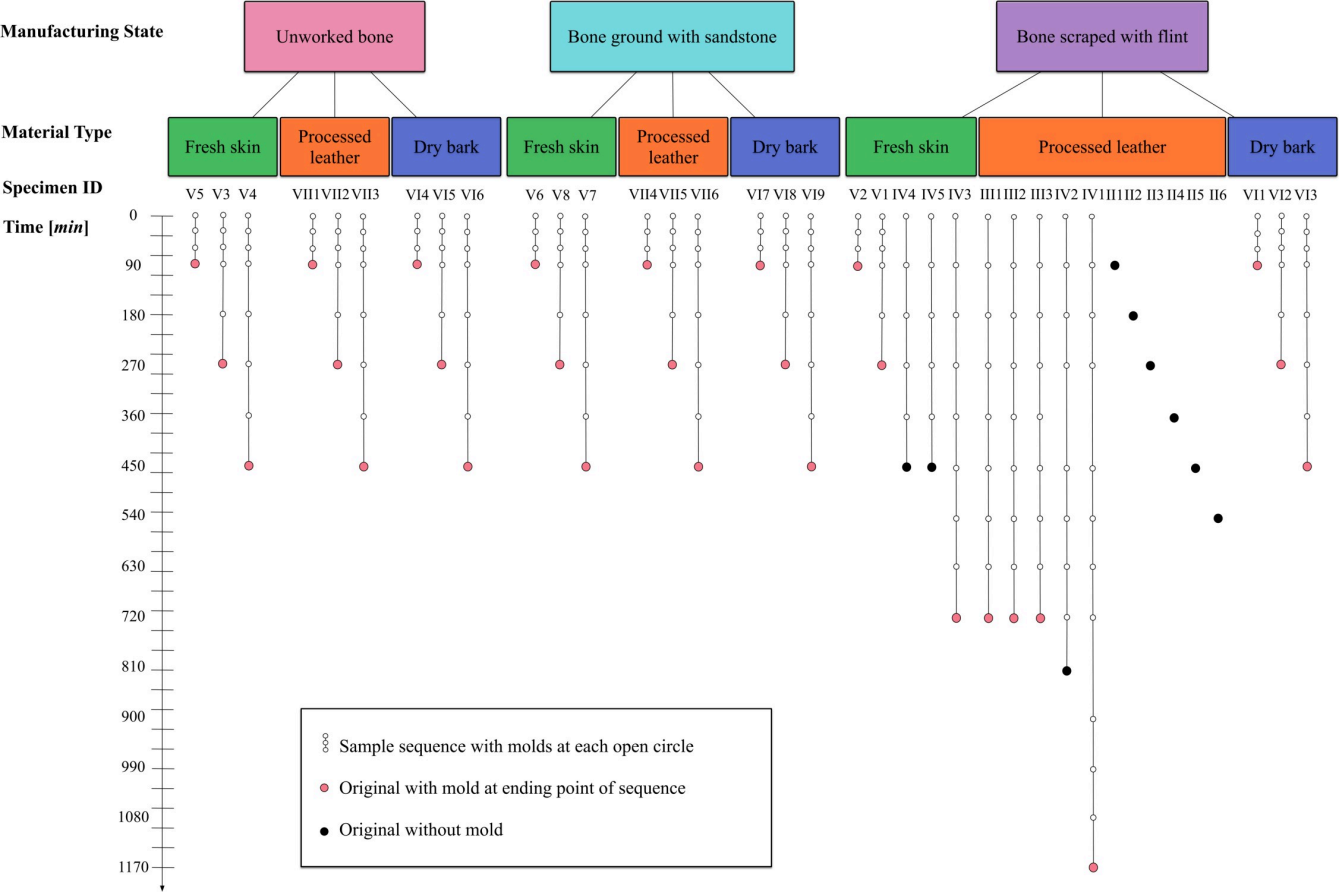
Schematic of experimental design. Specimen information including *Manufacturing state*, *Material type*, *Time* interval when bone surface mold was taken, and total duration of *Time* of each bone in the experiment.

Our study assesses how bone wears during extended use on three *Material types* (fresh skin, processed leather, and dry bark), starting from three *Manufacturing states* (unworked, modified by sandstone to grind or by flint to scrape) over *Time* by taking incremental molds of bone specimens subjected to a controlled, mechanical experiment. A total of 37 bones (*Specimens*) and 202 bone surface molds were produced, providing 239 total samples (Fig 1). Each sample was then scanned at least five times using a confocal disc-scanning microscope. 3D surface texture analysis (comparable to [43–45]) was used to quantitatively measure various features of bone surfaces using ISO 25178 parameters, such as surface roughness [*Sa*], autocorrelation length [*Sal*], peak curvature [*Spc*], and upper material ratio [*Smr1*]. Then, we employ a multilevel multivariate Bayesian model to explain parameter variation under experimental conditions.

### Experimental design

**Fig 2:**
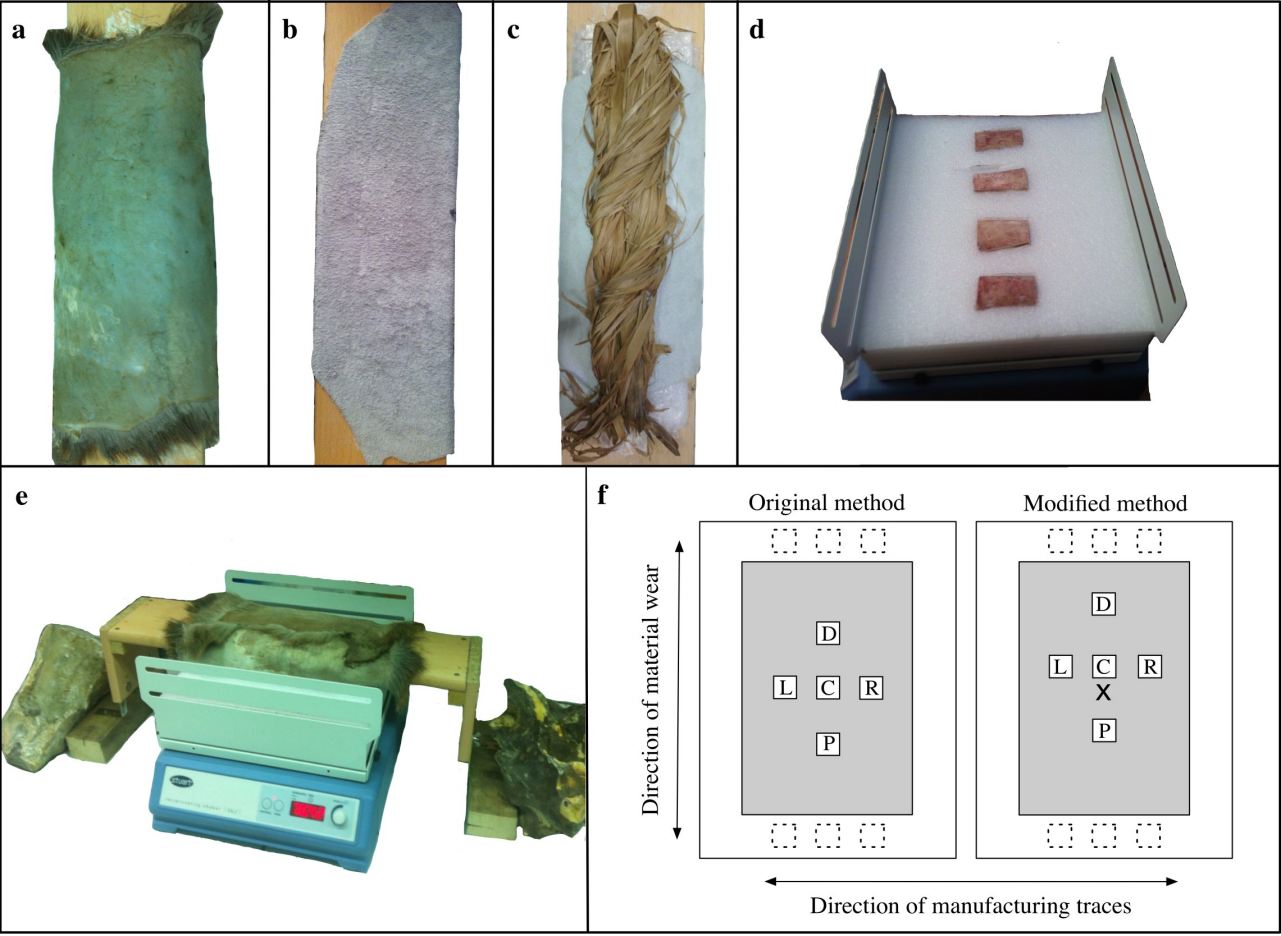
Setup of experiment and scanning locations. (a-c) Material setup on external platform; (a) fresh skin; (b) processed leather; (c) dry bark; (d) bone specimens inserted into foam on a Stuart SSL2 rocking machine (bones in this image are from a test sample with placement in the foam perpendicular to the direction of all the bones in the subsequent experiment); (e) platform with material over the Stuart SSL2 in the experimental position; (f) examples of crosswise sampling using two methods for obtaining scans at locations C (center), D (distal), P (proximal), L (left), and R (right). Additional scanned locations are represented by dashed boxes at the extreme distal and proximal ends. All scanned areas are 0.8mm^2^ but are enlarged here for readability. Modified sampling method uses an incised “x-mark” for finding the center over all samples.

To approximate the repetitive motion of bone used on a particular material, we obtained a reciprocating shaker (Stuart SSL2, Stone, Staffordshire, UK). Bone specimens were placed on the shaker, which moved them back and forth. A stationary wooden frame over the shaker held the material to rub the bone specimens, which were inserted into a foam block (Fig 2). The shaker was used to control systematically for time, motion, and strokes per minute during the experiment. The machine has a 2 cm range of motion and can be set to rock back and forth from 25 to 250 strokes per minute. We set the machine to 180 strokes/min and found that running the machine any faster made the entire system unstable. A timer on the machine allowed the experiment to run at intervals up to 90 minutes at a time (Fig 2).

Cow (*Bos taurus*) ribs were obtained in five separate *Lots*. The majority derived from fresh cuts of *plat de côte* (the sternal portion of beef ribs) originating from four separate trips to several butchers in Paris, France, while defleshed, partially fresh, cow ribs were obtained from Archeoshop, a vendor selling items specifically for archaeological experimental purposes. No animals were slaughtered for the express purpose of these experiments. Fresh ribs were kept in a refrigerator for approximately one week and partially fresh ribs were left at room temperature for no more than two weeks before experiments were conducted. We do not have information on how long these specimens were aged before butchery or, in the case of Archeoshop, kept after defleshing. Recent research has suggested that storage type and length of storage may be a factor affecting bone freshness and degradation [73, 74], though these factors have not been tested for bone surface modifications.

The ribs from the butcher shops were cleaned manually by NLM with flint flakes. Meat was removed; the periosteum was not intentionally removed, but some may have come off during the cleaning process. Care was taken to limit the amount of traces left on the surface in this stage, however, if the periosteum had been removed, this may have left traces on the bones creating uneven starting surfaces. The Archeoshop ribs did not have flesh on them, and therefore they were used as is. It is possible that some of the periosteum was removed during their cleaning, but this is unknown to us. A hacksaw with a 30 cm steel blade was used to manually saw the ribs into rectangular shapes with most dimensions ranging between 3 and 5 cm (S1 Table). Of these bone pieces, 37 of the flattest *Specimens* were chosen to ensure even working surfaces that could be positioned flush with the abrasive material fixed to the wooden frame over the shaker. Irregular edges and surfaces were removed before the experiment using a metal file to ensure that the surfaces around the edges were angled downward. This was important, because these irregular surfaces might damage the material. Traces from the metal file are restricted to the edges and in the case that there are some present on the working area, the regular traces of the metal file should be distinguishable from the experimentally produced traces with flint and sandstone. Each bone was labeled with a specimen number on one of the sawn edges, which was then arbitrarily called the distal end. Depending on the experiment, bones at this point were marked with an incised X at the central location to be used as a visual landmark during the scanning procedure (S2 Table). Bones were divided into three categories for subsequent *Manufacturing states*: unworked, ground with sandstone, and scraped with flint. The bones that were unworked were examined to ensure that no traces were left from the butchering or cleaning process. All other bones were modified transversely to the grain of the bone. Modified bones were either scraped with a flint flake or ground with sandstone until the entire surface of the bone was altered to a uniform state (S2 Table).

Each bone *Manufacturing state* was used on three *Material types*: fresh skin (red deer, *Cervus elaphus*), processed leather (cow, *Bos taurus*), and dry bark (linden, *Tilia*). The processed leather, obtained from BHV (Bazar de l'Hôtel de Ville), Marais, Paris, was soft and supple and was cut into several pieces about 40 by 50 cm. The fresh skin, complete with hair attached but with membrane removed, was cut into smaller pieces of approximately 40 by 50 cm. The skin was soaked to remove preservative salt before experimentation began. The bark came in strips about 1 to 2 cm wide. Both the skin and bark were obtained from Archeoshop (Fig 2).

On the machine itself, we attached a stiff foam block with depressions specifically shaped for each bone; this allowed the flat surface of the bone to be exposed during the material-wear process (Fig 2D). Bones were placed longitudinally into the foam so that movement of the machine ran with the grain of the bone and most of the modifications were perpendicular to the movement. This allowed for modification and use-wear traces to be completely distinguishable. A removable and adjustable wooden exterior platform approximately 70 cm long, 20 cm wide, and 2 cm thick was built to lie across the machine. The materials that were used to produce wear traces on the bone were centered on the underside of the platform to make contact with the bone surfaces (Fig 2A–2C and 2E). Each experimental material was affixed to the platform in slightly different ways. The processed leather was easily pulled tight and then affixed with water-soluble craft glue. The deerskin had to be pulled tight and then nailed to the side of the platform. The skin contained the hair, which made this material quite thick. We added extra bulk between the platform and the skin by using hair from other parts of the skin to ensure that this material had the same amount of thickness over the entire working area (~4 cm thick). Since the bark was in thin strips, we decided to twist the strands together into one bunch. One side of this bunch was then affixed to the platform with water-soluble glue. The other side was free from any effects of the glue. The platform was fairly wide so that a large surface area of material could be used on the bones in the rocking machine. This allowed for a maximum of six bones to be run at the same time (Fig 2).

Bone surface molds were taken using dental impression materials (similar to [75])(President Jet Plus Light Body, Coltène, Altstätten, Switzerland). Molds were made before each experiment, at 30-minute intervals for the first 90 minutes of most of the experiments, and at 90-minute intervals after that depending on the length of each experiment (Fig 1). These molds served as controls and allowed us to obtain a record of the changes in the bone surfaces through time. Before molds were taken, each bone was cleaned with 70% ethanol to ensure that any particulate matter that could adhere to the bone microtopography was removed. We followed cleaning protocols recommended for bone surfaces as described by Bromage [76] and Martinez et al. [77]. We avoided stronger solvents, because they could cause permanent damage to the bone surface [76]. We visually inspected each specimen to ensure that cleaning had been successful. This procedure was especially important for the bone specimens used with fresh skin and dry bark, both materials that deposited particulate matter on the bone. After the bone was removed from the mold, we replaced it in the apparatus and experimentation resumed.

### 3D surface scanning

Each original bone *Specimen* and the bone surface molds were scanned at multiple loci with a confocal disc-scanning microscope (μsurf mobile, Nanofocus AG, Oberhausen, Germany) using a 20x lens (numerical aperture = 0.4, field of view = 0.8 mm^2^). Because of its large working distance (12mm), the 20x lens has the advantage of covering a large field of view and allowing for large sample curvature (large δz variation). Scan quality was reviewed after each scan was completed. Scans with 95% or more of the surface measured were accepted. Those of lesser accuracy were recaptured with alterations to the pitch, gain, exposure, or brightness until 95% of the surface was captured.

At least five surface scans per sample were taken with the confocal disc-scanning microscope. To ensure that this portion of the methodology was replicable, five scans on each bone were laid out in a crosswise formation with one scan in the center, two along the longitudinal axis, and two along the horizontal axis (Fig 2F). Each bone was first measured for length and width at the midsection of the bone (S1 Table). Then, 2 cm were subtracted from the total measurements (1 cm from each edge) to define the central scanning area. The first scan was taken in the exact center of this area. The other four scans were taken in a crosswise formation exactly halfway between the center scan and the limit of the center scanning area (S1 Table). For recording purposes, the five scans were labeled C, D, P, L, and R, signifying Center, Distal, Proximal, Left, and Right, respectively (Fig 2F).

Two methods were used to ensure that each bone surface mold was scanned in the same location as its bone counterpart. First, the C location on the bone was marked with a felt-tip pen after being scanned. Each mold was then placed back onto the bone in its original location so that some of the marker transferred to the mold to obtain the C location. In some cases, it was also possible to find a visual marker at the C location to ensure accuracy. The scans D and P were determined in the exact same way as was done for the bone, but the scans L and R were reversed because the molds are mirror images. After using the felt-tip method for finding the C location of each mold, it became apparent that some of the marker did not absorb into the mold, causing some inaccuracy in the scans. An attempt was made to remove the liquid marker stain, while retaining the location of point C. This proved to be a difficult task, so the bones that were run in all subsequent experiments were subject to a second procedure for identifying and recording the center location. These bones were marked with an incised X before any experiments were run or molds taken (S2 Table). This step ensured that all molds would have a consistent reference point. Since the incised X was the center of the bone surface, each of the five scans was shifted distally by 2.7 mm. This was achieved by visually centering the μsurf mobile instrument on the incised X and then moving 2.7 mm distally. From this new C location, the four other locations were determined as before (Fig 2F). The five scans from each sample are within the central scanning area, so the altered location of these scans should not significantly affect the results.

**Fig 3:**
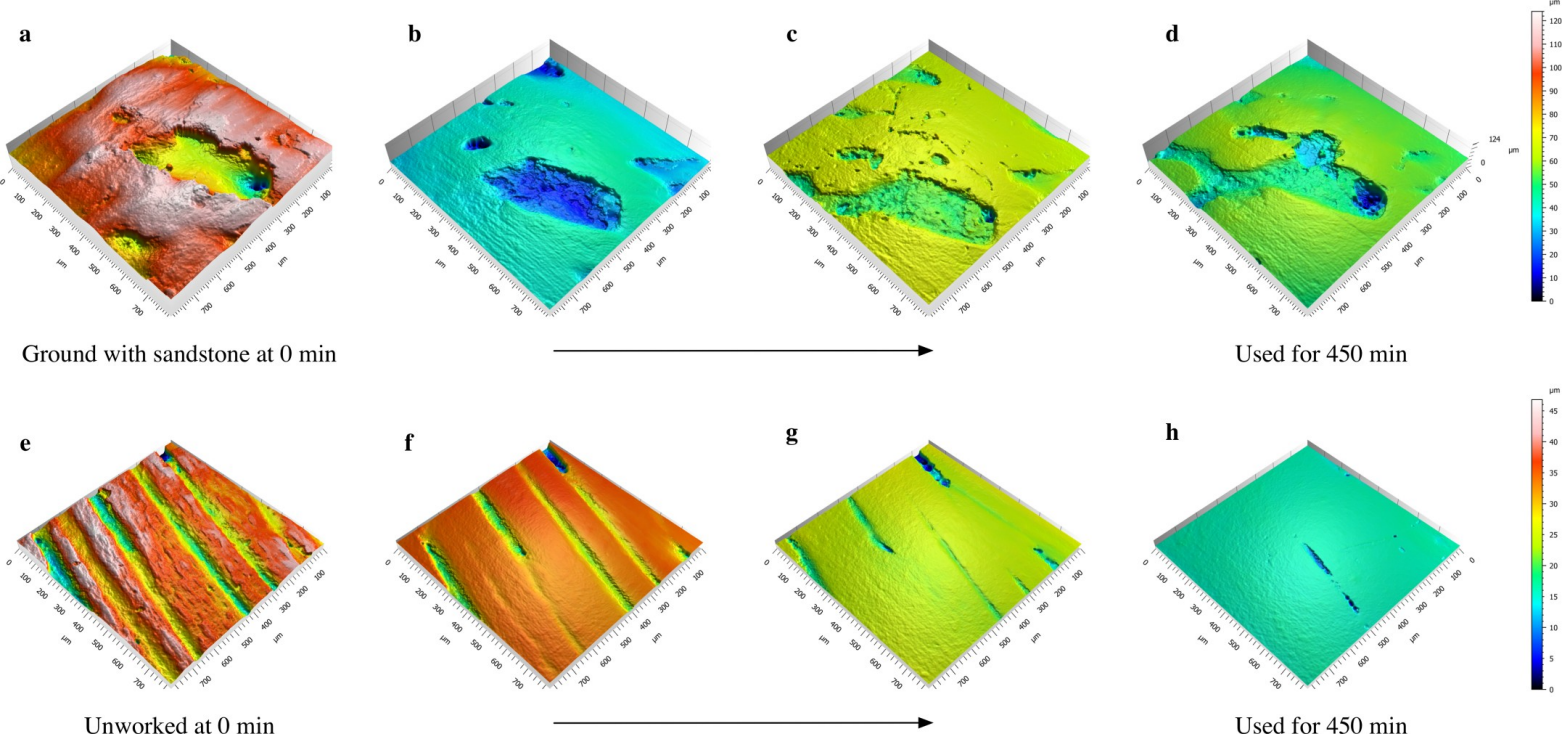
Sequence of meshed axiomatic 3D models. Images show the effect of fresh skin on a highly porous bone initially modified by grinding (a-d) and on an unworked bone (e-h) over time. Meshed axiomatic 3D models in each row are taken from molds from the same specimen and in roughly the same location (note the slight differences in scanning locations for (a-d). (a) 0 min; (b) 90 min; (c) 180 min; (d) 450 min; (e) 0 min; (f) 60 min; (g) 180 min; (h) 450 min. Color corresponds to height on the z-axis with red representing high elevations and blue the lowest.

It should be noted that there might be slight differences in the position of the scans from mold to mold within a sequence or with respect to the original bone sample. All precautions were taken to obtain similar positioning, and in many cases, it was possible to visually assess the accuracy of the scanning location. There are some cases where this was not possible because the visual cues were ambiguous or had changed significantly from the previous scan. In these cases, we relied on the distances obtained from our initial measurements (S1 Table). In the cases where it is clear that each successive scan is not in precisely the same location as the previous scan, this will only affect analyses that focus on a singular scanned location through time (Figs 3A–3D). Since we are attempting to understand how bone surface changes with action from different materials, assessing the variation of many scanning locations will be most useful.

To make the experiment standardized and repeatable, the sampling method focuses on the defined central scanning area. This defined area may not be the actual major surface area worked, since each bone exhibits a slightly different curvature. In particular, there may be distinct differences in bones with concave and convex surfaces. To make the experiment completely standardized, each bone specimen would have to be derived from the same location on a particular element (of animals of the same size, age, etc.). This was neither cost-effective nor logistically possible, so we opted to use the flattest portions of the cow ribs. While these pieces are fairly flat, there is still enough variation to cause distribution of wear to be slightly different between specimens. During the scanning procedure, we noticed that some of the scanned locations were insufficiently worn, so we scanned extra points on many of the samples to obtain scans for a variety of surface wear states for each experimental scenario (S3 Table). Most of the additional scans come from locations near the distal and proximal edges of the bone (Fig 2F). If the bone is slightly concave, the edges will be the surface portions that are in most contact with the material. If a bone is completely flat, the bone first comes into contact with the material at the edges, and this is where the most pressure occurs during each pass of the bone. Because these additional scans were not in the original experimental design, we did not use them to fit the statistical model, but our empirical findings of the non-modeled scans will be discussed.

### 3D surface texture analysis

We applied metrological pre-processing to each of the bone surface scans to reduce the nominal form by filtering long-scale components of the surface (waviness) from short-scale components (surface roughness and noise) following ISO 25178 recommendations for technical surface scans. Translated to bone as a biological surface, long-scale components are bone curvature, form, and shape, while short-scale components are wear traces and measurement noise. Following successful applications and testing of different pre-processing techniques in tooth wear studies [45, 78], we use a similar combination of a filter and an operator to reduce measurement noise applying a low pass S-Filter and a F-Operator as form removal. We applied the following procedures in Mountains Map Premium v. 7.4.8076 Analysis software by Digital Surf (Besançon, France) using *operators* with the following specifications (in brackets): *leveling* (least square method), *mirroring* (the y- and z-axes) in case of molds, and *outlier removal* (outlier removal method: removal of isolated outliers and those around edges, with normal strength, and fill in of holes <225 points, removal of noise), *fill in of non-measured points* (smoothing method calculated from neighbors). Each non-measured point is replaced by a value obtained as compared to the neighboring valid points. The F-operator *remove form* was set using a polynomial of second order (polynomial of degree 2).

**Fig 4:**
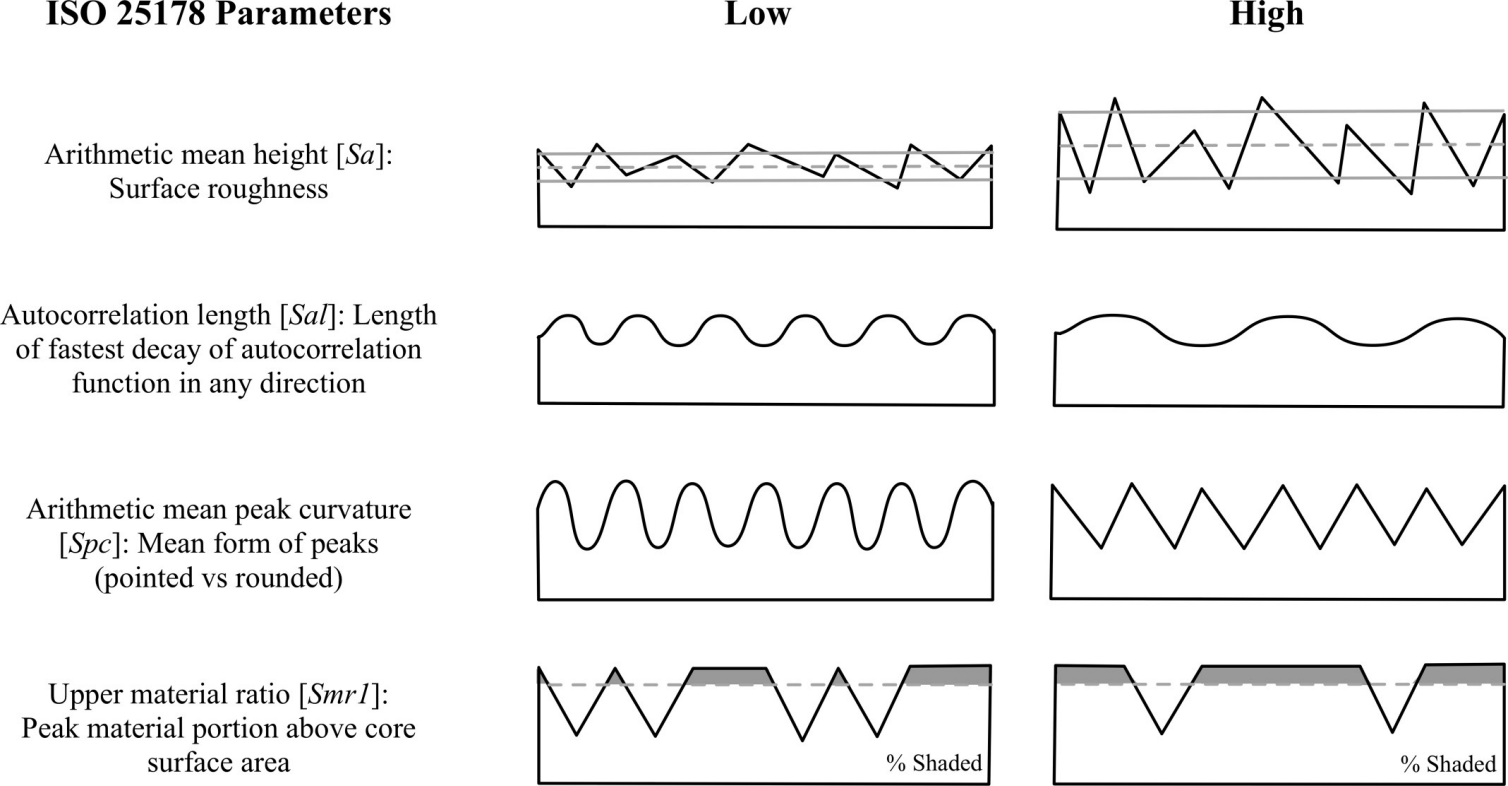
2D depictions of ISO 25178 3D texture parameters. Illustrations on the left show examples of surfaces with low values, while those on the right have high values (adapted/modified from [79]).

From the meshed axiomatic 3D models, we chose the following four of the 30 ISO 25178 parameters for statistical modeling: arithmetic mean height [*Sa*], autocorrelation length [*Sal*], arithmetic mean peak curvature [*Spc*], and upper material ratio [*Smr1*] (Fig 4). 3D surface texture parameters were used to obtain an overall understanding of the surfaces in all experimental states and were chosen to be representative for the four main parameter groups of the ISO 25178 as well as potentially diagnostic of differences in surface features. We chose *Sa* as a robust representative for the height parameters indicating the statistical distribution of heights along the z-axis of a surface, *Sal* as representative for the spatial parameters indicating periodicity and directionality of a surface, *Spc* as representative for feature parameters indicating the geometry of particular segments of a surface (*e*.*g*., peaks), and *Smr1* as representative for the functional parameters indicating bearing function calculated from the material ratio curve. Initially, we checked all 30 ISO 25178 parameters. However, since many of the possible parameters are correlated, or even mathematically related to one another, we chose four parameters that were relatively independent, based on pairwise scatter plots.

### Statistical modeling of 3D surface texture parameters

The values of the four surface texture parameters are greater than zero for all observations, so a natural log transform was applied to stabilize variances and symmetrize distributions. The covariate *Time* (in minutes) was also log-transformed, after adding the value 1.0 to all observations. Cases for which log(*Time* + 1.0) = 0 then form a baseline for comparison of changes in surface texture over time.

The statistical model for the observations **Y**, a matrix having p = 4 columns (log-transformed surface texture parameters) and n = 1253 rows (meshed axiomatic 3D models), is a multivariate mixed model of the form

Y=XB+ZU+E,

(see *e*.*g*., [80]) where **XB** represents fixed experimental and control effects, **ZU** represents random effects, and **E** is residual error. Models of this type are applied in studies of animal breeding [81] and longitudinal growth [82]. In a recent study, Katz et al. [83] demonstrate an anthropological application. In our analysis, **ZU** captures idiosyncratic effects of bone *Specimens* and their *Lots* of acquisition which are not under experimental control but are needed for a full accounting of sources of variation. **U** is a 42x4 matrix of random intercepts, where each column of **U** contains 37 unique *Specimen* effects and 5 unique *Lot* effects. Each of the four surface texture parameters is represented by a column of **U**. **Z** is a 1253x42 matrix of zeros and ones, which associates the appropriate *Specimen*- and *Lot*-effects with each observation. The dimensions of **X** and **B** depend on the number of fixed effects in the model. For example, **B** would be a 2x4 matrix in a model containing a single binary control variable and an intercept for each surface texture parameter. The corresponding design matrix **X** would be a 1253x2 matrix of zeros and ones. For more complex models incorporating additional covariates, the row dimension of **B** and the column dimension of **X** would increase accordingly. For all models, **E** is a 1253x4 residual matrix.

The model took several control variables into account, aiming to avoid confounding and to sharpen inferences about experimental variables. One of these control factors is the *Location* of scans. The five sampling locations were chosen so that they would be representative of overall specimen transformation and could be easily replicated over each bone mold. Because these five locations were spread over a large portion of the bone, it is possible that the different locations were altered at different rates or in different ways. Assuming scans along each axis were transformed in similar ways, we grouped our sampling locations into three categories in the model: Center, Left and Right combined, Distal and Proximal combined (Fig 2F).

Other control variables in the model are *Bone-vs-Mold* and *Rescan test*. Scans were taken of each specimen and most of their molds at the end of each experiment (Fig 1). The scans of the molds should be exact mirror images of the scans of the bone at the same time interval; the model effect *Bone-vs-Mold* permits a check of this expectation. We also took additional scans of some molds one year after they were formed (S4 Table). We include *Rescan test* as a control variable in our model to determine if molds are distorted over a period of a year. Accuracy of the molds can then be assessed.

Preliminary modeling indicated that variation in surface texture parameters was over-dispersed with respect to the Gaussian distribution. We therefore chose the multivariate Student-t as the observation-level distribution. Random effects for *Specimen* and *Lot* are adequately modeled by multivariate Gaussian distributions, based on goodness of fit checks described below.

We fit four models of increasing complexity, starting from an “empty” model containing only random effects, then sequentially adding control covariates, experimental effects and interactions. We compared widely applicable information criterion (WAIC) scores [84] to investigate the predictive quality of each model (Table 1). WAIC is similar to the more familiar Akaike Information Criterion (AIC) [85], but is better suited to the computational approach we use. Based on the WAIC scores, the most complex model envisioned by the experimental design (M3) was used to generate model predictions. M3 is a multilevel, multivariate Bayesian model, including pairwise and three-way interactions of three experimental factors (*Material type*, *Manufacturing state*, and log *Time*), control factors (*Location* of scan, *Bone-vs-Mold*, *Rescan test* after one year), random effects (*Specimen* and *Lot*), and *Error* (Table 2).

**Table 1:** WAIC score differences relative to the score for model M3.

Model	Effects	Δ WAIC
**M0**	random	531
**M1**	random + control + experimental (time only)	336
**M2**	random + control + experimental + pairwise interactions	33
**M3**	random + control + experimental + pairwise interactions + 3-way interaction	0

Model M3 has the lowest WAIC score and is therefore preferred for out-of-sample prediction—the standard for model quality measured by WAIC. Larger WAIC score differences indicate less preferred models relative to M3. The models increase in complexity reading down the table.

**Table 2:** Effects included in model M3 and their representation in the model’s design space.

MODEL COMPONENTS		DESIGN MATRIX
fixed effects	control factors	location + bone-vs-mold + rescan test
	experimental factors	+ log time + manufacture
	pairwise interactions	+ manufacture*log time + material*log time
	3-way interaction	+ manufacture*material*log time
random effects and error		+ lot + specimen + error

We used independent Gaussian priors with mean zero and standard deviation 5.0 for model parameters describing experimental and control effects (elements of **B** in the multivariate mixed model). Priors for variance/covariance matrices of random effects (**U**), as well as for the scale matrix of multivariate Student-t errors (**E**), are specified using decompositions of the form **Σ** = **D** Ω **D**, where **Σ** is a variance/covariance or scale matrix, **D** is a diagonal matrix and Ω is a correlation matrix. We used a half-Cauchy prior with scale 2.5 for the elements of **D** and an LKJ prior with parameter 1.5 for Ω [86]. This specification was used for random effects of *Specimen* and *Lot*, as well as for the multivariate Student-t *Error*. We used a Gamma prior with shape 2 and rate 0.1, truncated on the left at the value 2, for the degrees-of-freedom parameter of the multivariate Student-t distribution.

We estimated effects in the statistical model by a Hamiltonian Markov-chain Monte Carlo method, implemented in the library *rstan* version 2.15.1 [87] of the statistical computing language R version 3.4.1 [88]. We allowed a warmup of 500 iterations for each of two chains and subsequently generated 500 parameter samples per chain, to obtain a total of 1000 posterior samples for inference. We examined trace plots of model parameters and used convergence checks implemented in *rstan* to ensure mixing was adequate for inference. We examined scaled and squared Mahalanobis distances between observations and their predicted values to check goodness of fit. These distances were compared to theoretical quantiles of the F-distribution [89] by use of a quantile-quantile plot (S1 Fig).

## Predictions

**Fig 5:**
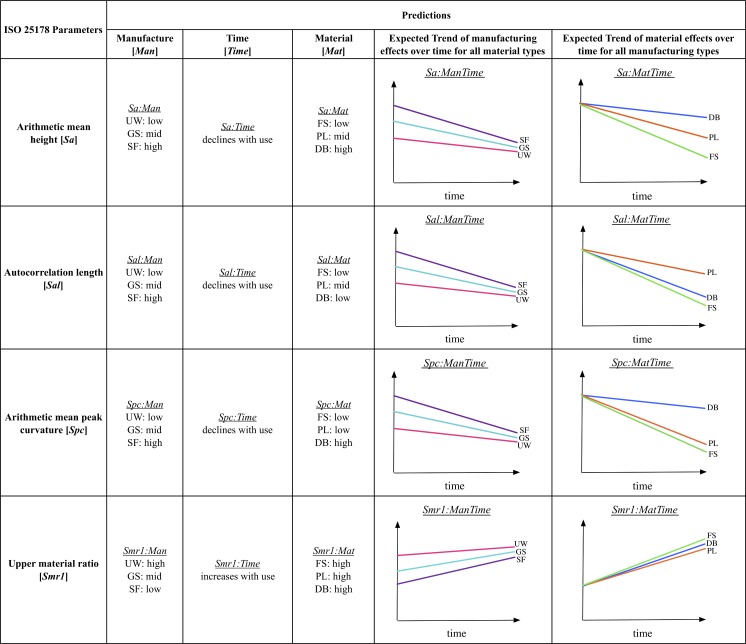
Predictions related to *Manufacture state*, *Time*, and *Material type* for the ISO 25178 parameters. UW: unworked, GS: ground with sandstone, SF: scraped with flint, FS: fresh skin, PL: processed leather, DB: dry bark. Figures show predicted trends of *Manufacturing state* effects (pink: unworked; turquoise: ground with sandstone; purple: scraped with flint) over *Time* for all *Material types* and predicted trends of *Material type* effects (green: fresh skin; orange: processed leather; blue: dry bark) over *Time* for all *Manufacturing states*.

Bone tissue is, by nature, variable in its texture and structure, and as a result, bone specimens are expected to display some variability in wear patterns. Microscopic bone will often exhibit uniform surfaces with only the finest bone structures visible (*e*.*g*., parallel fibers and lamellae) [90], but some surfaces may be more porous than others with large voids or small holes to accommodate blood vessels (Fig 3). These bone pores will affect the uniformity of unworked as well as any modified or used bone surfaces. Regardless, we can generate predictions about bone surface alterations related to use based on the design of the experiment. The experimental bone specimens start from three different *Manufacture states*: unworked, ground with sandstone, and scraped with flint. These three states should comprise varying features that can be quantitatively differentiated. Likewise, the properties of the three materials used on the experimental bones should be reflected by variation in the traces produced during use. Using dental mold material, molds were taken of each specimen over the course the experiment. This allowed us to address predictions specifically related to wear over *Time*. This experiment combined each *Material type* with each of the three *Manufacture states*, resulting in nine different manufacture-material interactions. Here, we discuss the basic predictions related to the three main factors of the experiment (*Manufacture state*, *Material type*, and *Time*) without taking any manufacture-material interactions into account. These predictions will be tested using arithmetic mean height [*Sa*], autocorrelation length [*Sal*], arithmetic mean peak curvature [*Spc*], and upper material ratio [*Smr1*] (Figs 4 and 5).

**Fig 6:**
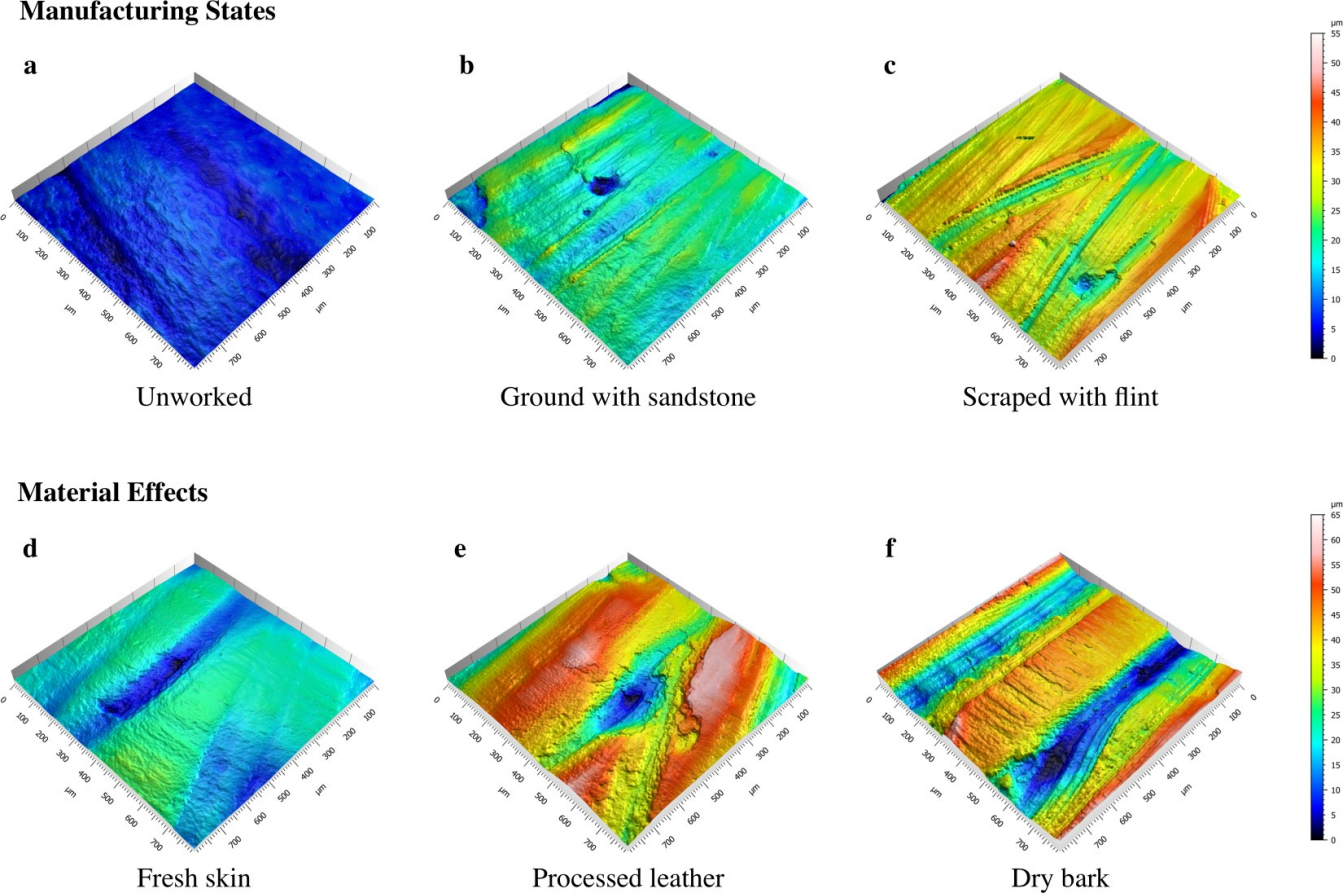
Meshed axiomatic 3D models. Images show *Manufacturing states* at time 0 of the experiment (a-c) and *Material type* effects at time 180 of the experiment (d-f); (a) unworked bone; (b) ground with sandstone; (c) scraped with flint; (d) fresh red deerskin; (e) processed cow leather; (f) linden bark.

Each bone specimen started from one of three mutually exclusive *Manufacturing states*: unworked, ground with sandstone, or scraped with flint (Fig 6A–6C). The sharp edges of flint create deep furrows in the bone surface; therefore, scraped bone should be rougher than both ground and unworked bone. The action of grinding often mimics that of scraping, and, therefore, ground bone should also be rougher than unworked bone (Fig 5, *Sa*:*Man*). Because modification to the bone surface causes fairly deep furrows, both scraped and ground bone should exhibit longer wavelength surfaces, while unworked bone should have a shorter wavelength. Since flint uses an edge to scrape and sandstone uses a surface to grind, the scraped bone should have the longest wavelength or low frequency surface (Fig 5, *Sal*:*Man*). The deep furrows created by grinding and scraping should cause the bone surface to be more varied with less material in the peaks. This effect should be lessened on ground bones due to the more uniform nature of the contact between the bone and grinding surfaces. Unworked bone should be more uniform and have a higher proportion of material in the upper peaks (Fig 5, *Smr1*:*Man*). Scraping with flint should result in many striations and sharp peaks between the striations. The small grains of the sandstone should have a similar effect, and cause relatively sharp peaks. Unworked bone should have the most rounded peaks (Fig 5, *Spc*:*Man*).

All experimental specimens were analyzed at the same loci at various time points in order to predict how time affects bone surfaces (Fig 1). For the materials tested here, we expect that constant abrasion will eventually cause the manufacturing traces or any original bone surface features to wear completely so that the surface is fully transformed (Fig 3H). Fig 5 displays hypothetical trends for each surface texture parameter over *Time* by *Manufacturing state* and *Material type*. At time 0, we predict that all the *Manufacturing states* should show varying values. As the experiment progresses, the surface of the bone should be worn in a manner unique to that specific material, and eventually the surfaces should converge on a value that is different from all starting values (see *e*.*g*., Fig 5, *Sa*:*ManTime*, *Sa*:*MatTime*). Peaks should wear at different rates depending on material, creating plateaus that will eventually wear down (Fig 5, *Smr1*:*Time*, *Smr1*:*MatTime*). Wavelengths of the bone surfaces should transition from high to low over time (Fig 5, *Sal*:*Time*). Regardless of the material used on each bone specimen, surfaces should become less rough as each experiment progresses (Fig 5, *Sa*:*Time*, *Sa*:*ManTime*). In addition, the surface peaks should become more rounded, but with variation dependent on material used (Fig 5, *Spc*:*Time*, *Spc*:*MatTime*).

Within each *Manufacturing state*, bone specimens were subject to one of three *Material types* (fresh skin, processed leather, or dry bark) (Fig 6D–6F). In the initial stages of the experiment, the bark-worn specimens should exhibit more surface roughness than either those used on fresh skin or processed leather, because bark is relatively rigid, and should wear only on the uppermost surfaces, leaving the furrows largely unchanged (Fig 5, *Sa*:*Mat*). This trait should also cause the surface wavelength to be higher in the bark-worn specimens. In subsequent stages of the experiment, the surface wavelength of bark- and fresh deerskin-worn surfaces should be lowest, because the abrasive nature of these materials should transform the bone surfaces more readily than those used on processed leather (Fig 5, *Sal*:*Mat*, *Sal*:*MatTime*). The flexibility of the skin and leather should allow the materials to wear on all surfaces, which should result in rounding of any high areas. Since bark is a rigid material composed of cellulose, it should cause peaks to have sharp edges (Fig 5, *Spc*:*Mat*, *Spc*:*MatTime*). All materials should wear bone surfaces creating increasingly large plateaus until the surface is smoothed (Fig 5, *Smr1*:*Mat*, *Smr1*:*MatTime*).

## Results

### The effect of manufacturing states

**Fig 7:**
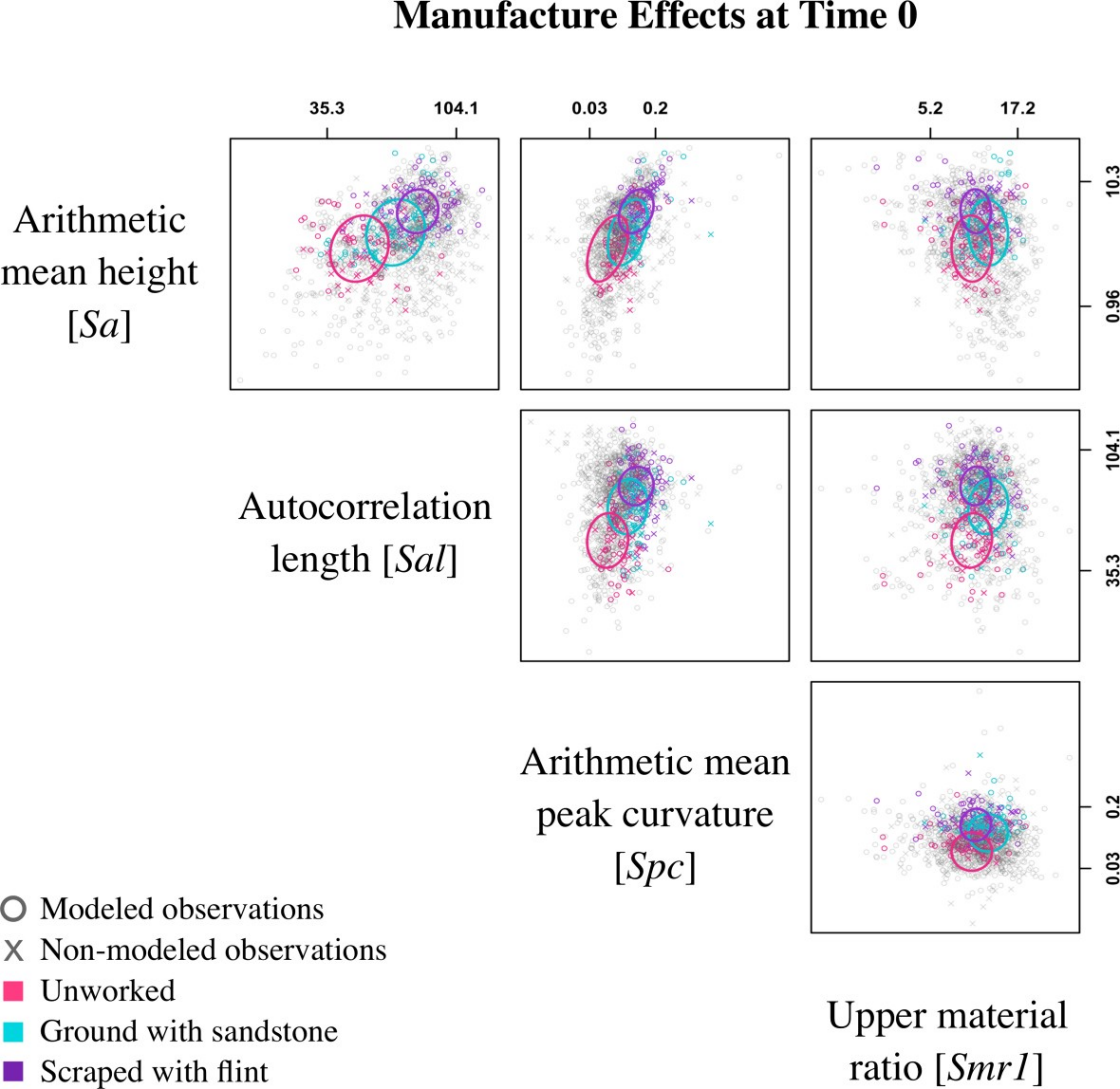
Scatterplot matrix at time 0. Plot shows all observations (o: modeled points; x: non-modeled points) displayed in the pairwise space of parameters: surface roughness [*Sa*], autocorrelation length [*Sal*], peak curvature [*Spc*], and upper material ratio [*Smr1*]. Colored observations are representative of *Manufacturing state* (pink: unworked; turquoise: ground with sandstone; purple: scraped with flint) at time 0 and ellipses show the model predictions of the mean for each pair of parameters using ellipse version 0.3–8 [91]. Axes are on the log scale, but tick labels are in original measurement units and placed at the 5th and 95th percentiles.

*Manufacturing state* is the only experimental effect on the samples at time 0. A scatterplot matrix displays all observations in the pairwise space of surface texture parameters, and the colored ellipses show approximate 95% posterior contours for the estimated means of each pair of parameters, for each of three *Manufacturing states* (Fig 7). Although there is no discrete separation between the three states, some trends are apparent. For the most part, these align with our initial predictions (Fig 5). In many of the comparisons, scraped bone is distinct from unworked bone while ground bone displays intermediate values. This is clearest in the comparison between *Sa* and *Sal* (Fig 7). Unworked bone has the lowest values for both surface texture parameters, while scraped bone has the highest values and ground bone is intermediate. This shows that bones modified by both flint and sandstone are rougher and have the longest wavelengths or lowest frequency surfaces. A similar pattern is evident in the comparison between *Sal* and *Spc*, which displays the lowest *Spc* values for unworked bone, indicating the most rounded surface peaks, and the highest *Spc* values for scraped bone. Our displayed model estimates (Fig 7) also show that ground bone has higher values for *Smr1* compared to scraped and unworked bone implying that ground bone has a larger portion of material in the peaks of the surface, though this parameter is not as clear and shows a considerable amount of overlap. In addition, *Smr1* is the only surface texture parameter that does not align with initial predictions for *Manufacturing state* (Table 3; Fig 5, *Smr1*:*Man*).

**Table 3:** Results of experiment in relation to *Manufacture state*, *Time*, and *Material type* for the ISO 25178 parameters.

ISO 25178 Parameters	Results		
	Manufacture [*Man*]	Time [*Time*]	Material [*Mat*]
**Arithmetic mean height [*Sa*]**	*Sa*:*Man* UW: low GS: mid SF: high	*Sa*:*Time* declines with use for FS; ***no clear trend for PL and DB***	*Sa*:*Mat* FS: low ***PL*: *high*** DB: high
**Autocorrelation length [*Sal*]**	*Sal*:*Man* UW: low GS: mid SF: high	*Sal*:*Time* ***no clear trend ***	*Sal*:*Mat* ***no clear differences***
**Arithmetic mean peak curvature [*Spc*]**	*Spc*:*Man* UW: low GS: mid SF: high	*Spc*:*Time* declines with use	*Spc*:*Mat* FS: low ***PL*: *mid*** ***DB*: *mid***
**Upper material ratio [*Smr1*]**	*Smr1*:*Man* ***UW*: *low*** ***GS*: *high*** ***SF*: *mid***	*Smr1*:*Time* increases with use for FS; ***no clear trend for PL and DB***	*Smr1*:*Mat* FS: high ***PL and DB variably lower***

Boxes outlined in bold include results that differ from predictions. UW: unworked, GS: ground with sandstone, SF: scraped with flint, FS: fresh skin, PL: processed leather, DB: dry bark.

### The effect of time

**Fig 8:**
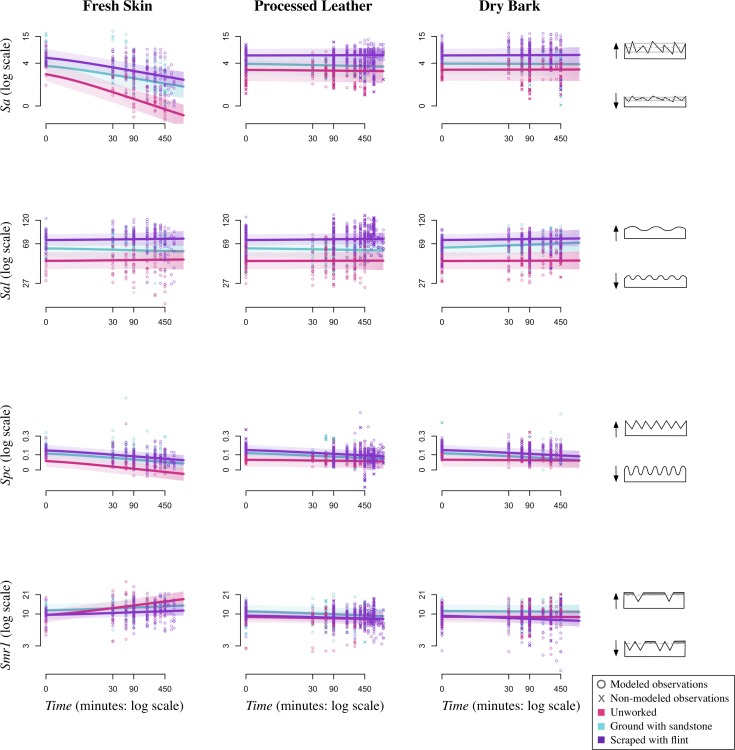
*Manufacture state* over *Time* by *Material type*. Plots showing the effects of *Manufacture state* over *Time* by *Material type* (fresh skin, processed leather, dry bark) and surface texture parameter (*Sa*, *Sal*, *Spc*, and *Smr1*). Lines show the model predictions of the mean for each *Manufacture state* (pink: unworked; turquoise: ground with sandstone; purple: scraped with flint). Both modeled and non-modeled points are displayed (o: modeled points; x: non-modeled points).

**Fig 9:**
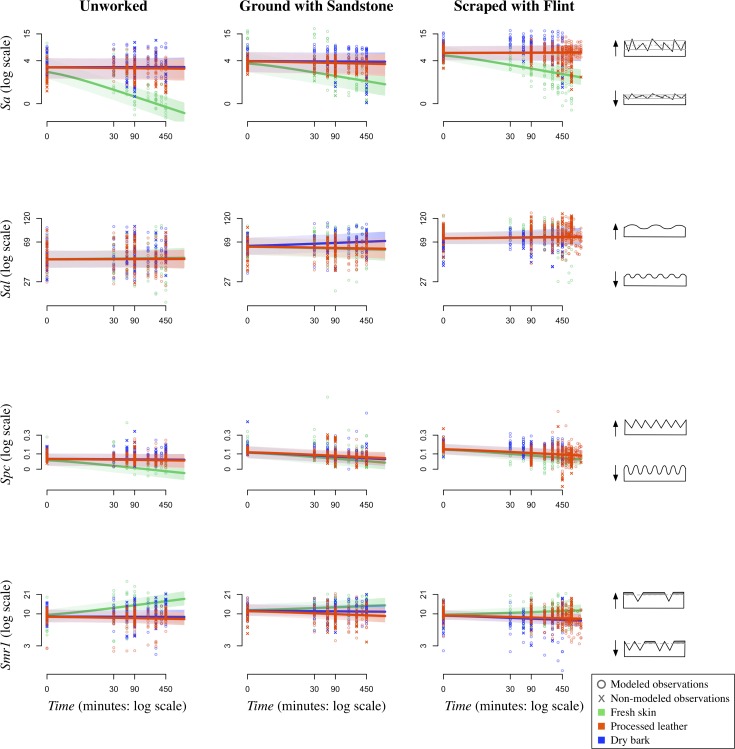
*Material type* over *Time* by *Manufacture state*. Plots showing the effects of *Material type* over *Time* by *Manufacture state* (unworked, ground with sandstone, and scraped with flint) and surface texture parameter (*Sa*, *Sal*, *Spc*, and *Smr1*). Lines show the model predictions of the mean for each *Material type* (green: fresh skin; orange: processed leather; blue: dry bark). Both modeled and non-modeled points are displayed (o: modeled points; x: non-modeled points).

The measurement of time-dependent effects of material use on bone is a unique aspect of our experiment. Because we took molds at the beginning of the experiment and at regular intervals throughout, we include *Time* as an experimental effect in our model. At time 0, our model estimates follow our predictions that differing mean values would be observed for the three *Manufacturing states*, though there is much variation across all surface texture parameters (Fig 8). Our predictions that *Sa* (Fig 5, *Sa*:*Time*) and *Spc* (Fig 5, *Spc*:*Time*) would decrease and *Smr1* (Fig 5, *Smr1*:*Time*) would increase over time hold true for fresh skin, but our results disagree with our prediction that *Sal* (Fig 5, *Sal*:*Time*) would decrease over time (Figs 8 and 9). Instead, there seems to be no change in *Sal* showing that the surface wavelength is consistent across the experiment (Table 3, *Sal*:*Time*). In general, the clearest trends are observed for fresh skin in both graphic representations of the model (Figs 8 and 9).

Our prediction that all surface parameter values would eventually converge on a value specific to a particular *Material type* is unsupported (see *e*.*g*., Fig 5, *Sa*:*ManTime*). In most cases, our model shows that though there is a trend in a particular direction, parameter values are clearly influenced by their original *Manufacturing state*. For example, the predicted mean for fresh skin decreases in roughness [*Sa*] over *Time* for all manufacturing types, but the separation in values by manufacturing state at time 0 persists throughout the experiment and in some cases becomes even greater throughout the experiment (Fig 8). The only instance where initial *Manufacturing state* is not reflected in final values is for *Smr1* when fresh skin is used on unworked bone. These values increase at a much quicker rate than those on scraped or ground bone. This quicker rate of change for unworked bone is also observed for *Sa* (Fig 8).

Similarly, our prediction that for any initial *Manufacturing state*, surface texture parameter values would be distinguishable over *Time* based on material used is partially supported (see *e*.*g*., Fig 5, *Sa*:*MatTime*). Over time, the fresh skin samples show surface parameter values that are distinguishable from the processed leather and dry bark samples (Fig 9). Bones used on processed leather and dry bark are rarely altered and retain values similar to their *Manufacturing states* throughout the experiment. However, even though the change in parameter values over time is minimal, there are some differences in material effects that will be discussed below.

### The effect of material types

**Fig 10:**
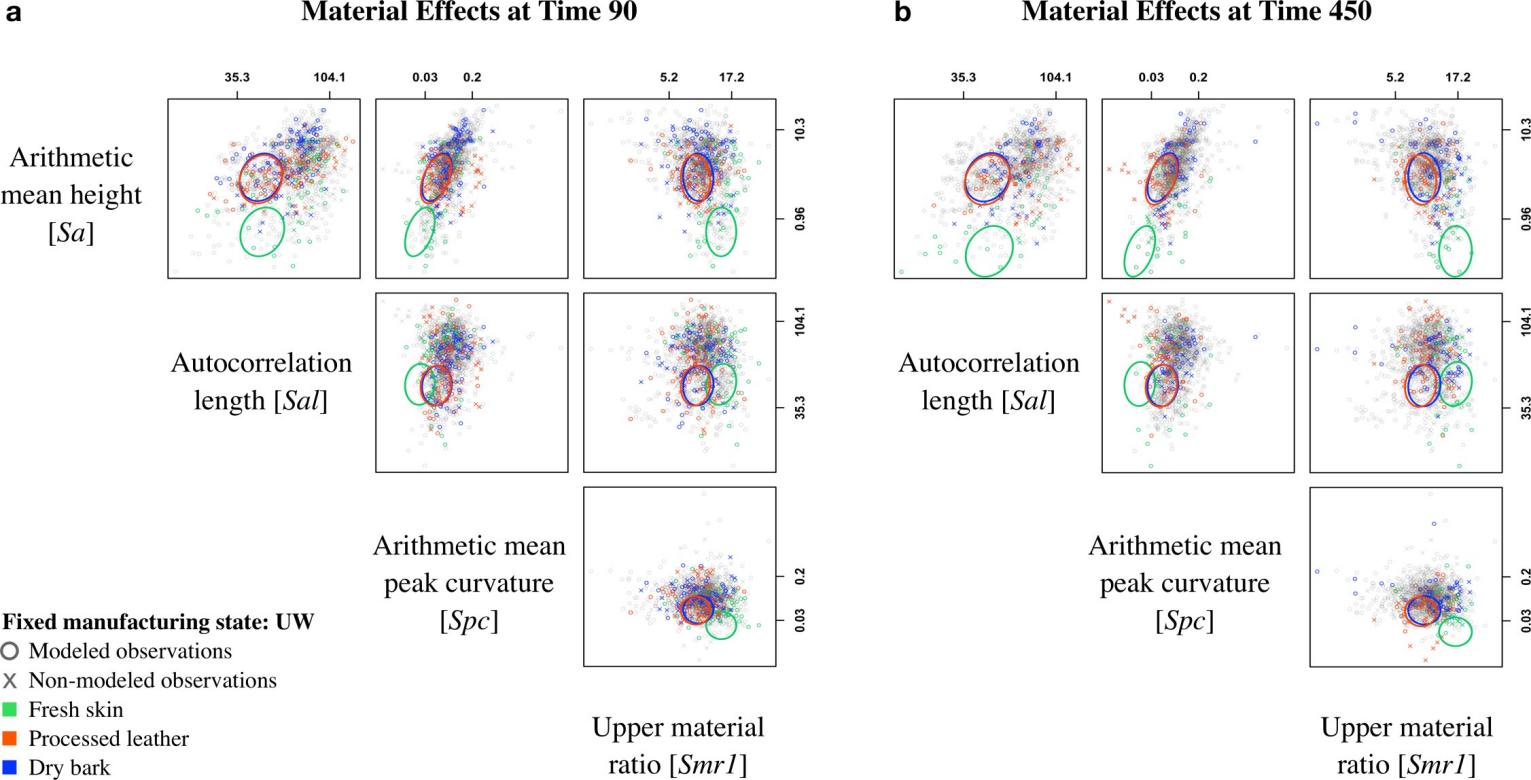
Scatterplot matrices at time 90 and 450. Plots show all observations (o: modeled points; x: non-modeled points) displayed in the pairwise space of parameters: surface roughness [*Sa*], autocorrelation length [*Sal*], peak curvature [*Spc*], and upper material ratio [*Smr1*]. Colored observations are representative of *Material type* (green: fresh skin; orange: processed leather; blue: dry bark) at (a) time 90 and (b) time 450 and ellipses show the model predictions of the mean for each pair of parameters using ellipse version 0.3–8 [91] for fixed *Manufacturing state*: Unworked (UW). Axes are on the log scale, but tick labels are in original measurement units and placed at the 5th and 95th percentiles.

Pairwise scatterplot matrices for four surface texture parameters show model estimates of the mean of two parameters at time 90 (Fig 10A) and 450 (Fig 10B), respectively, for each of the three materials (with *Manufacturing state* fixed at UW—unworked). For most comparisons at time 90, there is some overlap between the *Material types*, but at time 450 the fresh skin observations are more distinct. Our predictions that fresh skin would have the lowest values for *Sa* (Table 3; Fig 5, *Sa*:Mat) and *Spc* (Table 3; Fig 5, *Spc*:Mat) hold true, though there is considerable overlap with *Spc*. These results suggest that fresh skin samples have the most curved peaks [*Spc*] and are the least rough [*Sa*]. Conversely to our prediction for *Sal* (Fig 5, *Sal*:Mat), the model estimates show that the wavelength of the bone surfaces [*Sal*] may not be distinguishable by material type (Table 3, *Sal*:*Mat*). In addition, our model estimates that fresh skin produces surfaces that are more uniform with a highest percentage of material in the peaks (Table 3, *Sal*:*Smr1*). This disagrees with our predictions that all materials will produce surfaces with a high *Smr1* value (Fig 5, *Smr1*:Mat). In addition, our model disagrees with our predictions that processed leather and dry bark should be distinguishable (Fig 5).

Two additional scatterplot matrices indicate similar overall trends, and all comparisons show bone samples used on dry bark and processed leather to be undistinguishable. The first of these matrices (S2 Fig) fixes *Manufacturing state* at SF—scraped with flint, while the second (S3 Fig) fixes *Manufacturing state* at GS—ground with sandstone. In addition, estimated *Smr1* values for fresh skin are highest in every scenario, but these values are variable for the dry bark and processed leather samples.

### Modeled vs. non-modeled observations

Additional areas scanned (Fig 2F; S3 Table) for the purpose of obtaining a variety of surface wear states for each experimental scenario are represented by “Xs” in Figs 7–10, though these data are not included in the statistical model. As expected, the non-modeled points group with the modeled points at time 0 (Fig 7). These are baseline values, and no experimental effects related to *Material type* are present at this point in the experiment. As the experiment progressed, some differences between the modeled and non-modeled points were observed (Figs 8–10). This was most apparent for the surface texture parameter *Sa*. For all three *Manufacturing states*, the model estimates that fresh skin decreases in surface roughness to a greater extent than both processed leather and dry bark (Fig 9). Although this is true for the overall transformation of bone specimens, individual areas on some of the bone surfaces show differing results. The non-modeled points, represented by “Xs” in Fig 9, indicate that processed leather and dry bark can obtain *Sa* values within the range of fresh skin, though fresh skin tends to have the lowest *Sa* values in most cases.

Other parameters that may express differing values for modeled and non-modeled points are *Spc* and *Smr1* (Figs 9 and 10B). The non-modeled points for processed leather at time 450 and later in the experiment show low values for *Spc*, which indicates that bone worn with processed leather has exceptionally rounded peaks. Additionally, the trend for the dry bark worn specimens followed our initial prediction that *Smr1* values would increase as the experiment progresses (Fig 5, *Smr1*:*Mat*; Fig 10). This shows that the most worn surfaces of the bone specimens worked by both dry bark and fresh skin have the most uniform surfaces, with a large portion of material in the peaks of the surface. The processed leather non-modeled points had lower values than both fresh skin and dry bark. This suggests that processed leather produces less of a plateauing effect on the bone surface.

### Random effects and control factors

*Specimen* (each separate rib piece after cutting) and *Lot* of acquisition (each separate rib procurement event) were included as random effects in the model to determine if these factors contribute meaningfully to variation in surface texture. Intraclass correlations (ICC) for *Lot*, which measures the percentages of total unstructured variance attributable to *Lot*, range from 0.11 to 0.31 over the four surface texture parameters. Little of the variation is attributed to *Lot*, suggesting that neither the different storage methods of each *Lot*, nor differences between animals used in the experiment, substantially affect the microscopic bone surface. There is a similar amount of variation between each bone *Specimen* with a range in ICCs from 0.12 to 0.33, though the majority of unstructured variation is due to *Error* or the noise in the experiment.

Prior to running the model, we examined pairwise scatterplots of surface texture parameters for bones and matched molds at the end of each experiment (S4 Fig). Empirical observations of the scatter plots show that molds replicate the bone surfaces imperfectly, perhaps as a result of registration errors caused by slight frame-shifts when scanning the two different materials. Although we developed a protocol to designate the same locus on each bone and mold, human error likely contributes to differences in the scanning frame (Fig 3A–3D). In some cases, differences in surface parameters between bone and matched molds are quite large, indicating that variation exists not only across the specimens, but even in adjacent areas on the same specimen.

If all of the differences in the comparison of *Bone-vs-Mold* are registration errors, then we would expect the variation to scatter relatively equally above and below the 45-degree line (S4 Fig). Our empirical findings suggest errors of a more systematic nature for surface texture parameter *Spc* (peak curvature). *Spc* tends to be lower on the molds, indicating that they have more rounded peaks than their matched bones. This suggests that the dental mold material does not accurately replicate the smallest peaks occurring on bone.

Because additional scans were taken a year after the completion of all experiments, we attempted to replicate our original scans to assess if the molds had warped over time (S4 Table). Empirical observations of the scatter plots show that the molds had not warped or changed shape over the course of the year (S4 Fig). Both *Rescan test* and *Bone-vs-Mold* are included as control variables in our model. This ensured that the effects of the experimental factors—*Time*, *Manufacturing state*, and *Material type—*were corrected for potential confounding effects of the use of molds, or the warping of them over time.

## Discussion

### Manufacturing as homogenization process

Our results regarding *Manufacturing states* at time 0 of the experiment agree with our initial predictions with one difference. We accurately predict that unworked bone has the lowest values for *Sa*, *Sal*, and *Spc*, but our results for *Smr1* differ from our prediction (Table 3; Fig 5, *Smr1*:*Man*). In the context of this experiment, a high value for *Smr1* suggests that the bone surface is more uniform with a higher proportion of material in the plateaus. Our results, a lower value for *Smr1*, show instead that unworked bone has more varied surfaces with lower material portions in the peaks of the surfaces. This finding suggests that unworked bone is quite variable and the action of modifying it, using either flint or sandstone, often makes the surface more uniform (Fig 7). Even though the sharp edges of flint and particles in the sandstone produce deep furrows and high peaks, the peaks of the surfaces are often more uniform in their modifications. This is likely due to the fact that the surfaces with manufacturing traces were completely modified before each experiment with the purpose of attaining a homogenous working area. As expected, grinding bones produces *Smr1* values that are higher than those of scraped bone (Fig 5, *Smr1*:*Man*). This is likely due to the more uniform nature of the contact between the bone and grinding surfaces. One might expect that less thorough scraping and grinding of bone should produce a surface that is more variable with lower *Smr1* values.

### Time dependent transformation of the bone surface

This study has shown that duration of use is one of the most important factors affecting the transformation of the bone’s surface. However, because bone specimens were not transformed in consistent ways between and within specimens, it will be difficult to apply experimental results related to duration of use directly to archaeological artifacts. Nevertheless, bone worn for longer time intervals has more extreme use-wear characteristics than bone worn over shorter time periods (Fig 3E–3H), indicating low wear rates and proving the durability of bone as a raw material. Our experiment has shown that many features of the bone’s surface change over time, and it would be worthwhile to extend either the force or the duration of future experiments to achieve higher wear rates and more extreme use-wear characteristics. Nonetheless, moderate to extensively worn surfaces, especially in the case of bone specimens used on fresh skin, are completely distinguishable from the surfaces at time 0 of the experiment. Given the durability of bone against wear, it may be unproductive to attempt to define a set of parameters representative of a particular wear pattern over all time periods. An additional confounding factor is that bone worn with different materials may show similarities at different time intervals. For example, a bone used for a short amount of time on fresh skin produces a similar *Sa* value compared to a bone that was used on dry bark for a longer time period. The application of texture parameters for distinguishing worked materials previously undertaken in qualitative use-wear studies must be made with caution, and a complete understanding of not only the materials but of the specific measurements used in the ISO parameters should first be achieved.

### Persistence of initial surface microtopography

Contrary to our prediction that all features of the bone at time 0 would be completely transformed throughout the experiment (Fig 5), our results show that material traces are greatly affected by the initial *Manufacturing state* (Fig 8). In previous qualitative studies, it has been recognized that initial surface topographies can affect wear formation and distribution [23, 92, 93]. In this experiment, the various materials seem to alter bone surfaces, regardless of initial *Manufacturing state*, in consistent ways. The one exception is in the case of unworked bone, which is altered at a faster rate than both ground and scraped bone. This might be due to the fact that unworked bone has a relatively even microtopography compared to the furrowed surface of scraped and ground bone. Furthermore, material used on a bone with manufacturing traces or natural features such as pores has to overcome those traces and features. In addition, material traces tend to be produced on a much smaller scale (~5–10μm in width) compared to manufacturing traces (~30–100μm in width), so the material wear traces tend to develop on the peaks of the bone surfaces first, and sometimes do not modify the deeper furrows. Even when material rubs on the furrow bottoms of the bone and removes the finer manufacturing traces, often the bone micromorphology present at time 0 remains. However, the mix of trace types (manufacture and material) on a bone surface may lead to challenges of equifinality in interpretation. This will be an important component that should be taken into account when studying archaeological remains.

### Fresh skin as an abrasive agent

Each material produces different effects on bone over *Time*. Fresh skin tends to transform the bone surface more quickly compared to both processed leather and dry bark. This difference can be explained by a number of factors. First, the experimental setup might be inadequate for testing the different materials in the same way. Each material is fundamentally different from the other, so their interactions with the mechanical setup could vary significantly. Even so, because the materials possess different physical properties, the results could be revealing the difference in the abrasive qualities of each material. These results could show that fresh skin is inherently more abrasive than both dry bark and processed leather.

A second factor that may affect the outcome of bone surface wear is the state of the materials. The fresh skin is unprocessed, while the leather and bark are already transformed and in a dry state. The process of transforming the fresh skin during drying may add to the abrasive quality. In addition, fresh skin is moist, which helps the material cover the entire microtopography of the cortical bone more extensively including the furrows. The combination of the moist material with the supple nature of animal skin can allow this material to penetrate more areas of the surface for transformation. In addition, the moist, sticky surface of the skin likely acts as a sponge, trapping external particles such as dirt or dust. In this experimental case, it is also possible that some of the salt used to preserve the hide was not adequately removed at the start of the experiment. Salt crystals are coarse and could increase the abrasive quality of the skin. Additionally, microscopic particles from the bone itself can affect how the bone specimen is worn. While bone is abraded, microscopic bone particles are removed from the bone surface. It is likely that these particles become incorporated into the fresh skin, contributing to the further abrasion of the bone surface. This collection of external particles is less likely to occur with the dry materials such as bark and processed leather, because they lack the moisture that allows the particles to be integrated.

### The importance of sampling locations: non-modeled observations

The model is based on scans from five locations within the central scanning area of the bone surface (Fig 2F). Therefore, the results of this experiment are broadly reflective of the surface topography across the bone specimens. By visual inspection, we determined that bone specimens were modified in different ways, and in many cases, the central scanning area was not significantly worn. Our model estimates likely reflect a procedure that failed to sample the most worn locations on each specimen, since the gross surface topography of each bone is distinct. It is difficult to draw firm conclusions about specific differences in how the *Material types* affect the bone surface using only the five modeled locations, so we discuss the empirical results of additional scanned areas not included in the statistical model here (Figs 7–10).

Non-modeled points indicate that processed leather and dry bark can obtain *Sa* values within the range of fresh skin (Fig 9). This result indicates that *Sa* is highly variable, that similar values can be produced through differing processes, and that time is a better indicator of reduced *Sa* values. In general, fresh skin tends to have the lowest *Sa* values, so even though there is a large amount of variation, it may be possible to distinguish *Material type* wear for the most extreme wear states.

When looking at the specific areas of the bone that are more transformed, some trends become apparent. The processed leather and fresh skin samples have the most rounded peaks of the surface [*Spc*], and the fresh skin and dry bark samples are more uniform with plateauing effects on the upper peaks [*Smr1*]. The rounded nature of the bone surface peaks is likely caused by the supple animal skins that wear on all the surfaces of the microtopography. Interestingly, bone worn with processed leather can have exceptionally rounded peaks (Figs 9 and 10B). In many cases these values are lower than those for fresh skin, which suggests that the two different states of animal skin exhibit different properties shaping the upper peaks of the bone surface. Contrary to our prediction that all materials would eventually show high values for *Smr1* (Fig 5, *Smr1*:*Mat*), the processed leather samples never reached the highest values, even for the non-modeled points. While fresh hide and dry bark have a more abrasive quality resulting in the plateauing of the surface (like scraped layers off of the surface), processed leather acts as a polisher or buffer. Less material is removed by the processed leather, so the microtopography of the surface reflects the initial state of the bone, but with rounding to all the surface portions. Alternatively, fresh skin rounds and abrades, while dry bark abrades the uppermost surfaces, removing material to create plateaus.

### Idiosyncratic variation

Though there are trends present at different stages of the experiment, a great amount of unexplained variation exists. Within an experimental setting, variation generally comes in two forms: signal and noise. Signal is experimentally induced and under the control of the experimental design. On the other hand, noise is a product of the heterogeneity between the experimental specimens. No matter how many variables are under control, certain features of the individual experimental units are idiosyncratic and unpredictable. Our results show low signal versus high noise. The signal is low because many of the surface texture parameters are minimally changed over the course of the experiment and the material effects are often similar between *Material types*. And even though we controlled a number of experimental variables, a high degree of noise still exists. This noise is likely due to the variable nature of bone. Bone is a porous material with natural fissures, but these features are not spread uniformly over the cortical surface of the bone. Many of these irregular pores are visible on the surface even after all of the manufacturing traces are removed, and in some cases, additional pores are exposed during the wear process (*e*.*g*., Fig 3C). A parameter that tests roughness [*Sa*] will pick up any inconsistencies in the bone and a porous specimen likely would not achieve a similar *Sa* value in a longer experiment as the other less porous ones. We tried to apply filtering algorithms to separate the shape of pores from manufacturing traces; however, this was not possible because sometimes manufacturing traces (signal) are a similar shape and wavelength scale as pores (noise). In the future it may be possible that machine-learning filtering algorithms could be developed to recognize the three-dimensional irregular pattern of bone.

### Limitations

In this study, we focused on understanding how bone is modified over *Time* on various materials; however, differentiating between *Material type* use on bone was only clear in the most extreme cases. Unexplained variation confounding our abilities to distinguish material wear traces may result from a number of factors. Here, we present limitations of our experimental design that may have contributed to this idiosyncratic variation.

The experiment was designed to assess the range of bone wear possibilities over time, and in doing that created a grade of measurable variables. In a scenario such as this, it may be impossible to locate any discrete variables. It was expected that *Material types* would become distinguishable as the experiment progresses, but some of our results suggest that the surfaces may become more similar with work regardless of material used. To assess this unexpected result, future studies could qualitatively define various states of wear during experimentation and then determine if material wear is distinguishable in those circumstances.

A great deal of variation produced in the experiment was due to the rather large cut bone specimens. The slightly different shapes of each bone resulted in different patterns of wear across the bone surfaces. This is commonly seen on bone tools, which exhibit the greatest amount of wear at the distal end with a weakening wear signature moving proximally from the distal end [29, 94]. To experimentally obtain more uniformly worn surfaces, smaller specimens with the same amount of curvature could help to ensure that the entire surface area is worn to similar extents.

Additionally, much of the unexplained variation in this experiment is likely caused by natural features of the bone, like fissures or pores. Many of the surface texture parameters are highly sensitive to the microtopography of the bone surface, so our measurements may not be completely reflective of the wear traces. A modified sampling strategy to avoid these natural features could be advantageous. At the scale of measurement used in this study, it may not be possible to avoid porous features. It could be beneficial to limit controlled experimental work to bones obtained from older animals whose boney structure has completed the growth process. Even so, future sampling strategies should employ a qualitative selection step to find the most appropriate areas for analysis. Once this area is defined, a quantitative sampling procedure will be important for repeatability. In addition, as discussed previously in *3D surface scanning*, steps should be taken to ensure that sampling across time sequences are taken in precisely the same locations. For example, a microscope with the ability to triangulate a repeatable 3D coordinate system across molds from the same bone specimen could be used to consistently locate points.

In addition, it is possible that some of the unexplained variation is a result of the microscope settings. We used a 20x lens with a numerical aperture (NA) of 0.4. An objective with a higher NA value should provide a higher resolution surface scan and theoretically, clearer results. A future study using a greater NA value could help resolve this issue.

This study represents an effort to integrate both prediction-based and controlled, replicable experimentation. We based our predictions of wear formation on previous qualitative observations that have informed many use-wear studies on bone [23, 25, 95–97]. Because use-wear analysis can be inconsistent and demonstrate unreplicable results between researchers [27], we designed an experiment to limit external factors and focus on the effects of variation in select independent variables. We predicted that material wear traces would be distinguishable over the course of the experiment, but even under controlled experimental conditions, our results were often ambiguous. Such results are consistent with previous qualitative attempts at distinguishing traces that are only determinable in the most extreme cases [27]. Several factors, including duration of use, bone morphology, manufacturing technique, and internal microstructure of bone must be considered when applying interpretations about tool use. Use-wear analysts must remain well aware of the issue of equifinality, the formation of similar observations through different processes, and how this limits our ability to interpret past behavior [98]. Our current understanding of trace formation may need to be reevaluated, and additional experimentation with the goal of better understanding bone wear processes should be undertaken. New methodologies should combine quantitative and qualitative use-wear analyses, both on surfaces and volumes. For now, our assumptions about the differences in plant and animal wear on bone should be questioned. When applying these types of analyses to archaeological remains, results should be presented with qualifiers and, if possible, with confidence intervals demonstrating the degree of certainty in the interpretation. Until further quantitative studies and standardized experimentation on bone tools are undertaken, functional interpretations of the use-wear traces should be made with caution.

### Recommendations for future 3D surface texture analyses on bone

Though our study provided tenuous results, it also provides a starting point for future quantitative studies to build upon. Our results indicate that there are consistent trends that demarcate the three *Manufacturing states* for surface texture parameters *Sa*, *Sal*, and *Spc* at time 0, while *Smr1* shows less distinguishable values (Fig 7). Although it is often possible to qualitatively distinguish manufacturing traces, a quantitative analysis can help bolster such determinations. In addition, this type of analysis may be used to analyze bone pieces that show ambiguous traces or, for one reason or another, are disputed formally modified bone tools [10]. For example, formal bone tool technology used by pre-modern human groups (*e*.*g*., [15]) is often highly contested and methodologies to study such pieces are held to higher standards than on those for studying modern human-made bone tools. The development of a quantitative methodology for determining if a bone tool has been manufactured will be very important in such cases. Our results indicate that there are clear trends distinguishing scraped from unworked surfaces, which is encouraging for future research.

To define the best methods and surface texture parameters to be used to study traces found on bone, it could be beneficial to work with manufacture and material traces separately. It might be the case that different parameters distinguish the different types of traces. From our experiment, *Sa*, *Sal*, and *Spc* show some differences for manufacturing traces, while *Sa*, *Spc*, and *Smr1* show possible distinguishing trends for material wear. Future researchers can build from this study and test additional parameters to refine our methodology. Once parameters for analyzing both manufacture and material traces are established, it may be possible to combine these findings into a larger model to distinguish all traces from each other.

## Conclusions

The controlled experiment and analysis presented here aimed to understand the basics of use-wear formation on bone over time and to develop a quantitative methodology for measuring the microtopography of bone surfaces, using principles from previous empirical use-wear analyses to create a more refined and effective approach. With this in mind, we designed our experiment to reduce external factors by using a mechanical setup in order to accurately assess the variation in a select number of 3D surface texture parameters. We chose to vary *Time*, *Manufacturing state*, and *Material type* under conditions that may be helpful for developing a broad understanding of manufacturing techniques and materials used in prehistory. Several trends were apparent that will provide a foundation for future work. *Time* is an important factor affecting the transformation of the bone’s surface and produces differing wear characteristics at short and long time intervals, which may obscure material wear patterns. Even so, unworked bone is completely distinguishable from bone used for long time intervals and those modified by scraping. The initial *Manufacturing state* of bone affects how bone wears and, in some cases, can be determined at the end of the experiment. *Material type* affects the rate of bone alteration and how it is transformed but does not often produce distinguishing traces. Our results indicate that even when a controlled experiment with reduced factors is performed, a great amount of unexplained variation exists most likely due to the variable nature of bone. Nevertheless, some of the trends observed here will be important to examine in future research. This study provides a starting point for investigations into basic characteristics of use-wear formation by exploring additional variables that are relevant to archaeological questions.

## Supporting information

S1 Table Measurement details of each specimen. Specimen details indicating measurements of the bone and between scanned locations (C: center, D: distal, P: proximal, L: left, R: right) with the Nanofocus.
(XLSX)

S2 Table Experimental details of each specimen. Specimen details indicating Manufacturing state and direction, duration of experiment, material worked, sampling method, and any additional notes.
(XLSX)

S3 Table Additional scans in selected locations on some specimens. List of additional scans taken in selected locations. Data from these meshed axiomatic 3D models are not included in the statistical model.
(XLSX)

S4 Table Additional scans for *Rescan test*. List of scans taken for the *Rescan test* one year after the molds were formed.
(XLSX)

S1 Fig Quantile-quantile plot of scaled and squared Mahalanobis distances. Mahalanobis distances (between observations and their predicted values) versus theoretical quantiles of the F-distribution (see [89] for details). The bulk of observations follow the theoretical quantiles well. The extreme point in the upper right corner depicts an observation that is *closer* to its predicted value than expected.
(TIF)

S2 Fig Scatterplot matrices at time 90 and 450. Plot shows all observations (o: modeled points; x: non-modeled points) displayed in the pairwise space of parameters: surface roughness [*Sa*], autocorrelation length [*Sal*], peak curvature [*Spc*], and upper material ratio [*Smr1*]. Colored observations are representative of *Material type* (green: fresh skin; orange: processed leather; blue: dry bark) at (a) time 90 and (b) time 450 and ellipses show the model predictions of the mean for each pair of parameters using ellipse version 0.3–8 [91] for fixed *Manufacturing state*: Scraped with flint (SF). Axes are on the log scale, but tick labels are in original measurement units and placed at the 5th and 95th percentiles.
(TIF)

S3 Fig Scatterplot matrices at time 90 and 450. Plot shows all observations (o: modeled points; x: non-modeled points) displayed in the pairwise space of parameters: surface roughness [*Sa*], autocorrelation length [*Sal*], peak curvature [*Spc*], and upper material ratio [*Smr1*]. Colored observations are representative of *Material type* (green: fresh skin; orange: processed leather; blue: dry bark) at (a) time 90 and (b) time 450 and ellipses show the model predictions of the mean for each pair of parameters using ellipse version 0.3–8 [91] for fixed *Manufacturing state*: Ground with sandstone (GS). Axes are on the log scale, but tick labels are in original measurement units and placed at the 5th and 95th percentiles.
(TIF)

S4 Fig Comparison of bone versus mold for four ISO 25178 parameters: *Sa*, *Sal*, *Spc*, and *Smr1*. Open circles are pairwise comparisons of the meshed axiomatic 3D models taken on bones and molds shortly after they were produced. Black dots are pairwise comparisons of bone and their molds scanned at least one year after they were produced. Lines represent equivalent ISO 25178 parameter values of the compared sample types. Axes are on the log scale, but tick labels are in original measurement units.
(TIF)
