# Supplementary figures and images for: Time wears on: Assessing how bone wears using 3D surface texture analysis
Source: PLoS One. 2018 Nov 7;13(11):e0206078. doi: 10.1371/journal.pone.0206078 (PMC6221309; doi:10.1371/journal.pone.0206078)

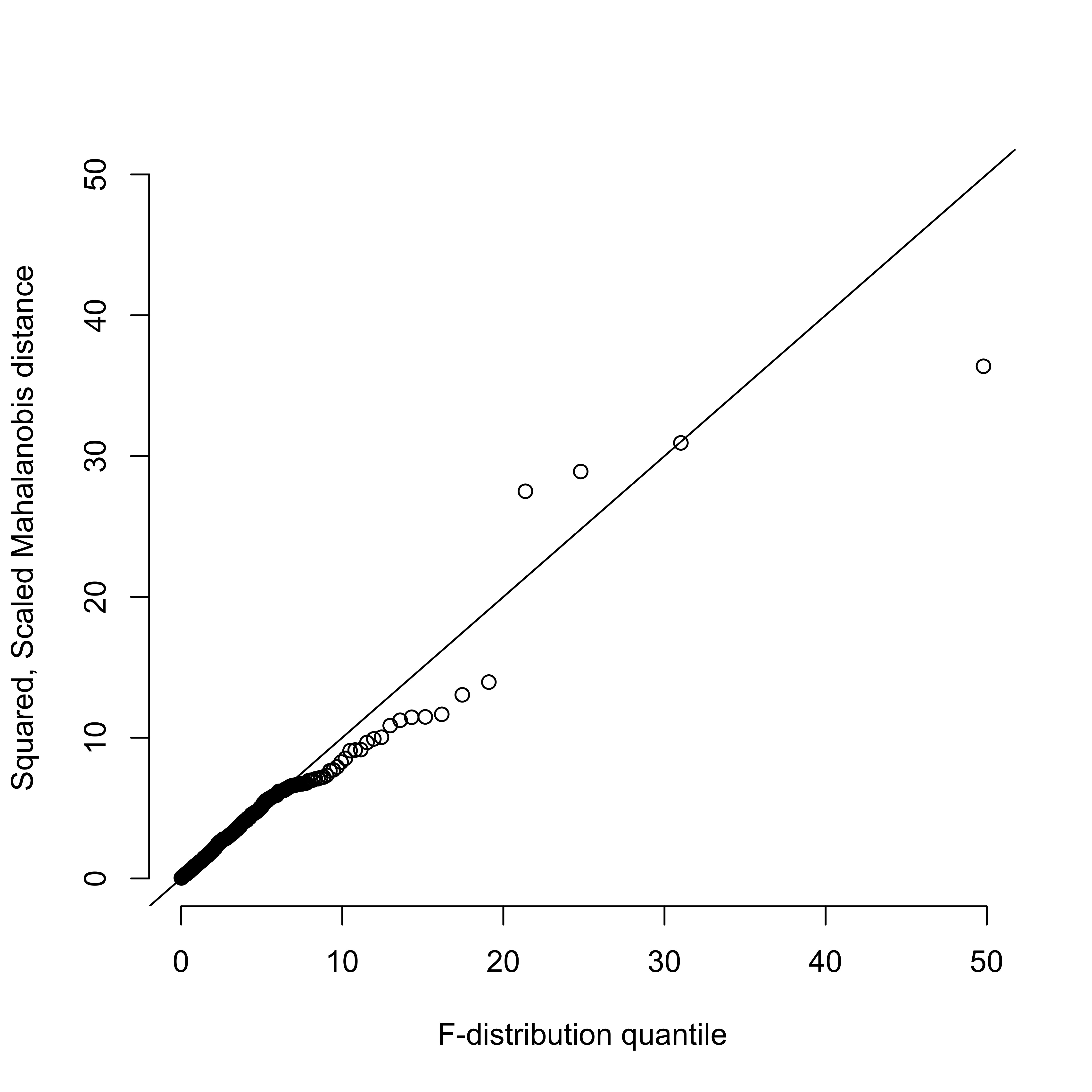

Supplement: S1 Fig — Mahalanobis distances (between observations and their predicted values) versus theoretical quantiles of the F-distribution (see [89] for details). The bulk of observations follow the theoretical quantiles well. The extreme point in the upper right corner depicts an observation that is closer to its predicted value than expected. (TIF) [file pone.0206078.s005.tif]

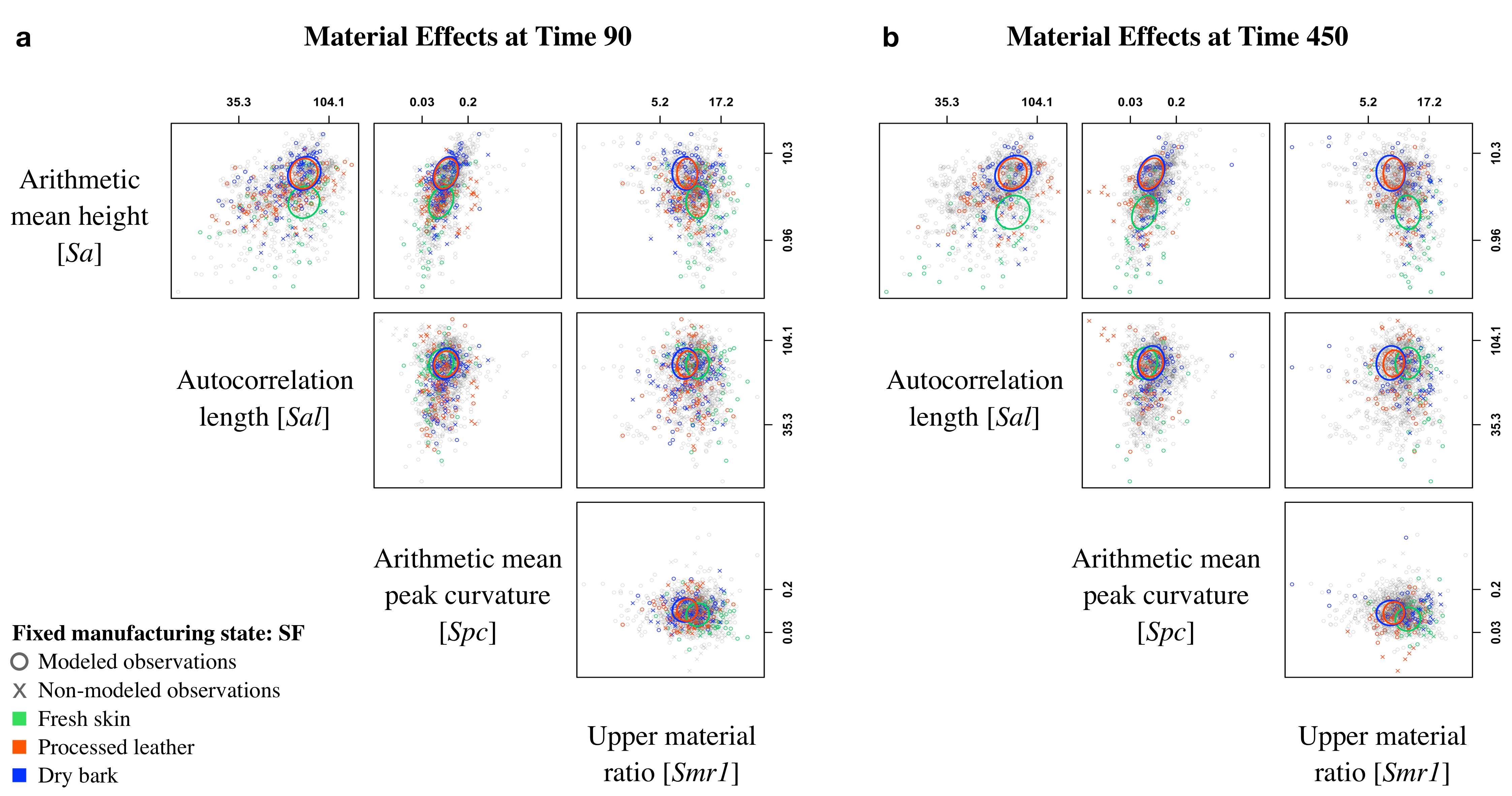

Supplement: S2 Fig — Plot shows all observations (o: modeled points; x: non-modeled points) displayed in the pairwise space of parameters: surface roughness [Sa], autocorrelation length [Sal], peak curvature [Spc], and upper material ratio [Smr1]. Colored observations are representative of Material type (green: fresh skin; orange: processed leather; blue: dry bark) at (a) time 90 and (b) time 450 and ellipses show the model predictions of the mean for each pair of parameters using ellipse version 0.3–8 [91] for fixed Manufacturing state: Scraped with flint (SF). Axes are on the log scale, but tick labels are in original measurement units and placed at the 5th and 95th percentiles. (TIF) [file pone.0206078.s006.tif]

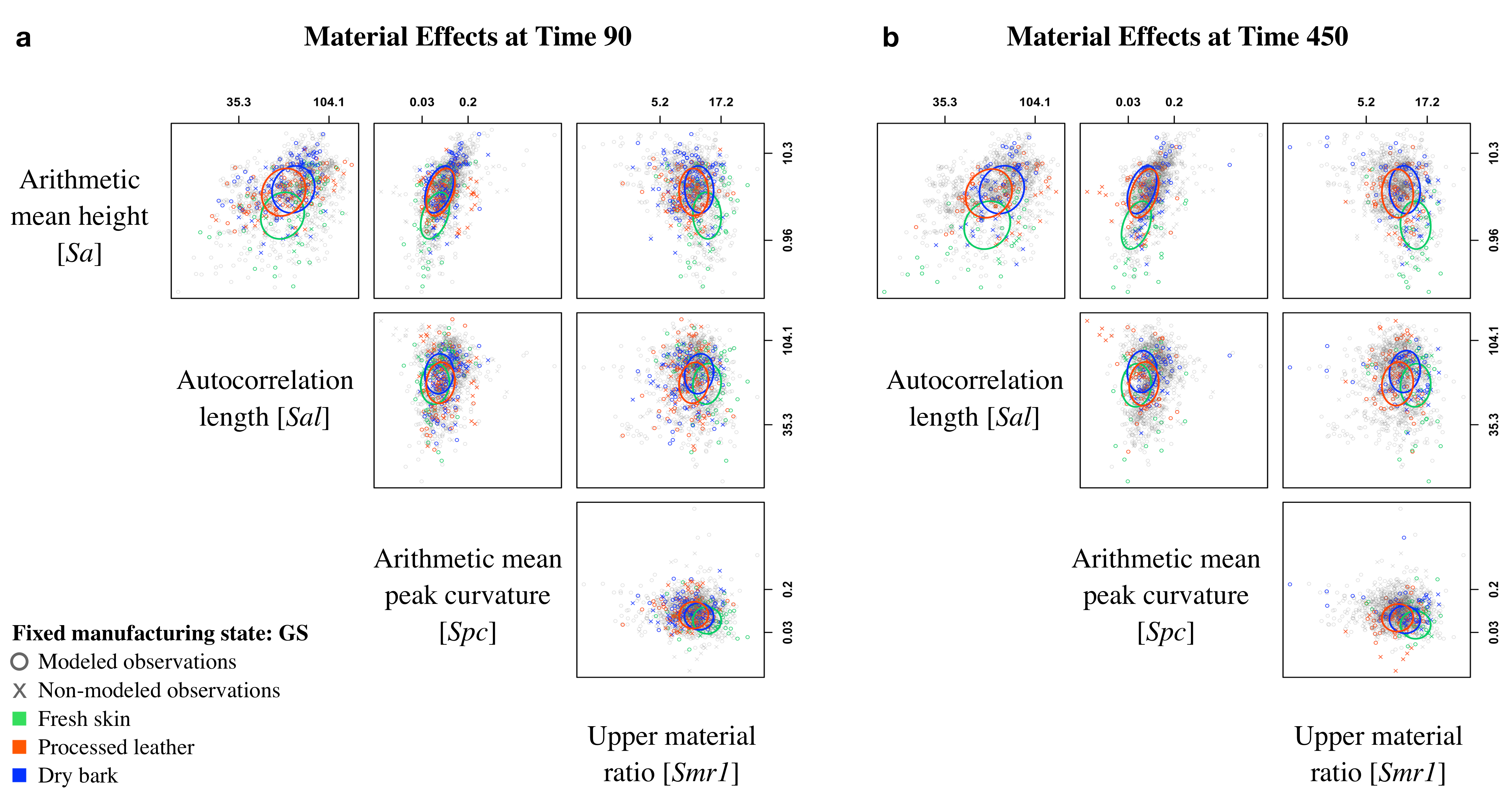

Supplement: S3 Fig — Plot shows all observations (o: modeled points; x: non-modeled points) displayed in the pairwise space of parameters: surface roughness [Sa], autocorrelation length [Sal], peak curvature [Spc], and upper material ratio [Smr1]. Colored observations are representative of Material type (green: fresh skin; orange: processed leather; blue: dry bark) at (a) time 90 and (b) time 450 and ellipses show the model predictions of the mean for each pair of parameters using ellipse version 0.3–8 [91] for fixed Manufacturing state: Ground with sandstone (GS). Axes are on the log scale, but tick labels are in original measurement units and placed at the 5th and 95th percentiles. (TIF) [file pone.0206078.s007.tif]

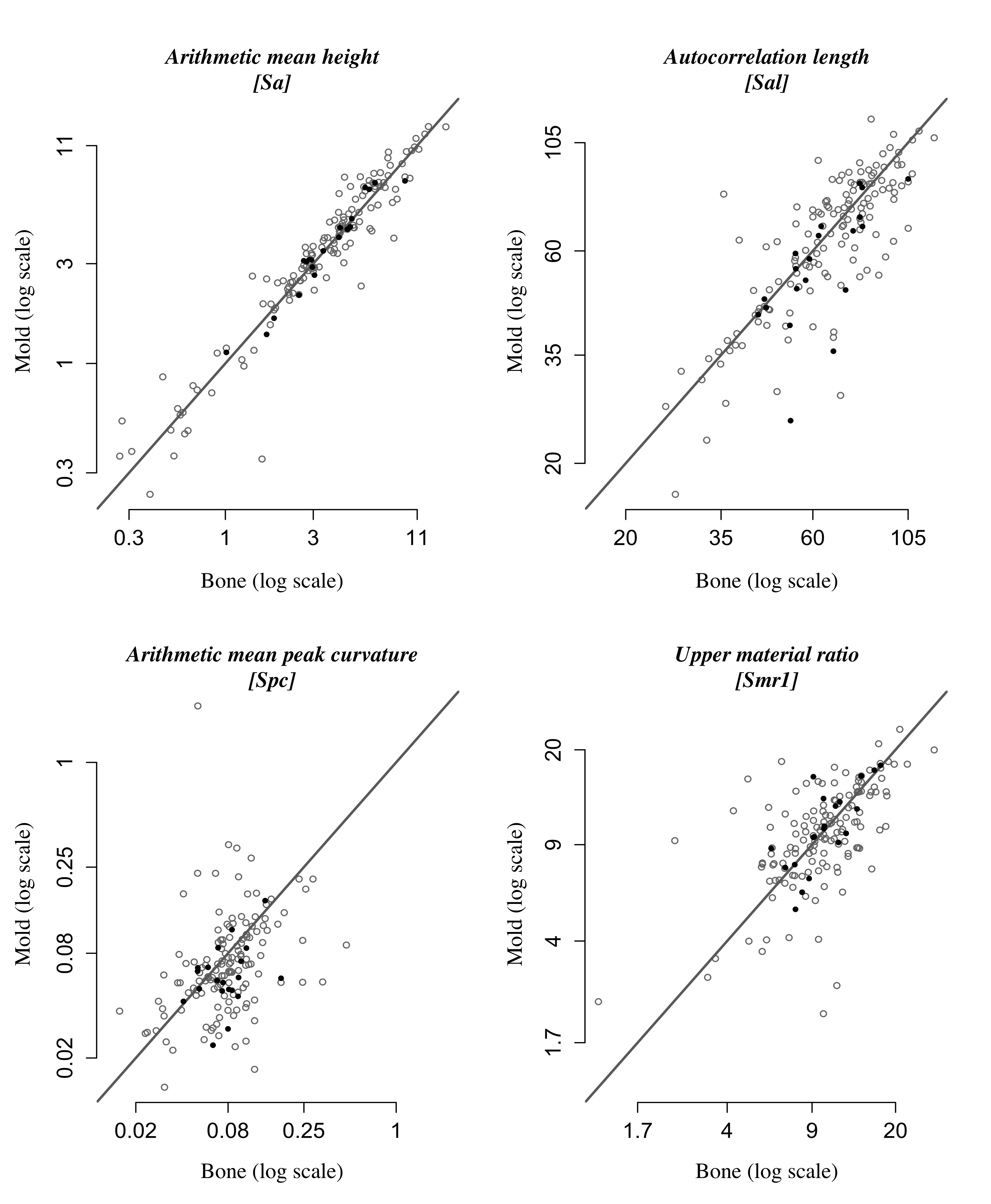

Supplement: S4 Fig — Open circles are pairwise comparisons of the meshed axiomatic 3D models taken on bones and molds shortly after they were produced. Black dots are pairwise comparisons of bone and their molds scanned at least one year after they were produced. Lines represent equivalent ISO 25178 parameter values of the compared sample types. Axes are on the log scale, but tick labels are in original measurement units. (TIF) [file pone.0206078.s008.tif]
